# Challenges Encountered When Evaluating an Antibody-Detecting Point-of-Care Test for Taeniosis in an Endemic Community in Zambia: A Prospective Diagnostic Accuracy Study

**DOI:** 10.3390/diagnostics11112039

**Published:** 2021-11-04

**Authors:** Chishimba Mubanga, Chiara Trevisan, Inge Van Damme, Veronika Schmidt, Isaac K. Phiri, Gideon Zulu, John Noh, Sukwan Handali, Richard Mambo, Mwelwa Chembensofu, Maxwell Masuku, Dries Reynders, Famke Jansen, Emmanuel Bottieau, Pascal Magnussen, Andrea S. Winkler, Pierre Dorny, Kabemba E. Mwape, Sarah Gabriel

**Affiliations:** 1 Department of Clinical Studies, School of Veterinary Medicine, University of Zambia, Lusaka 10101, Zambia or chishimba.mubanga@ugent.be (C.M.); igphiri@yahoo.co.uk (I.K.P.); gideonzulu@yahoo.com (G.Z.); richardmambo2@gmail.com (R.M.); malronc2003@yahoo.co.uk (M.C.); mmasuku69@yahoo.com (M.M.); evans.mwape@unza.zm (K.E.M.); 2 Department of Veterinary Public Health and Food Safety, Faculty of Veterinary Medicine, Ghent University, 9820 Merelbeke, Belgium; ctrevisan@itg.be (C.T.); inge.vandamme@ugent.be (I.V.D.); 3 Department of Biomedical Sciences, Institute of Tropical Medicine, 2000 Antwerp, Belgium; fjansen@itg.be (F.J.); pdorny@itg.be (P.D.); 4 Department of Neurology, Center for Global Health, Faculty of Medicine, Technical University of Munich, 81675 Munich, Germany; veronika.schmidt@tum.de (V.S.); andrea.winkler@tum.de (A.S.W.); 5 Centre for Global Health, Institute of Health and Society, Faculty of Medicine, University of Oslo, 0450 Oslo, Norway; 6 Eastern Provincial Health Office, Ministry of Health, Chipata 510023, Zambia; 7 Division of Parasitic Diseases and Malaria, Centers for Disease Control and Prevention, Atlanta, GA 30333, USA; jxn1@cdc.gov (J.N.); ahi0@cdc.gov (S.H.); 8 Department of Applied Mathematics, Computer Science and Statistics, Faculty of Sciences, Ghent University, 9000 Ghent, Belgium; dries.reynders@ugent.be; 9 Department of Clinical Sciences, Institute of Tropical Medicine, 2000 Antwerp, Belgium; ebottieau@itg.be; 10 Faculty of Health and Medical Sciences, University of Copenhagen, 2200 Copenhagen, Denmark; pma@sund.ku.dk

**Keywords:** *Taenia solium*, point of care test, diagnosis, taeniosis, copro Ag ELISA, copro PCR, rES33 EITB, sensitivity, specificity

## Abstract

*Taenia solium* taeniosis diagnosis is challenging because current tests perform sub-optimally and/or are expensive, require sophisticated equipment, infrastructure and trained manpower, and therefore are not community deployable. A recently-developed, multi-strip, *T. solium* point-of-care test (TS POC) for simultaneous detection of tapeworm (TS POC T) and cysticercus (TS POC CC) human antibodies was evaluated for diagnostic accuracy on consecutively recruited community participants in Sinda district, Zambia. All participants were tested using the TS POC test. All test-positives and 20% of the test-negative participants were invited to give a blood and stool sample for reference testing. Three different reference tests were used for taeniosis diagnosis: recombinant rES33 enzyme-linked immunoelectrotransfer blot (rES33 EITB), copro PCR and copro Ag ELISA. Bayesian analysis with probabilistic constraints was used to estimate sensitivity and specificity. In total, 1254 participants were tested with the TS POC test, of whom 13 tested positive using the TS POC T. Based on 161 participants with complete data, the estimated sensitivity and specificity for the TS POC T test were 38% (95% CI: 5–93%) and 99% (95% CI: 98–100%), respectively. The challenge of highly variable inter-assay performance is highlighted. We recommend either increasing the sensitivity or redesigning the test.

## 1. Introduction

*Taenia solium* taeniosis develops when humans harbor the tapeworm in the intestines. While taeniosis is often clinically inconspicuous, as the infected person rarely expresses symptoms except for mild abdominal discomfort, epidemiologically it is a very significant stage. Tapeworm carriers shed eggs that can infect people (and pigs) resulting in (neuro) cysticercosis, a disease with significant personal, public health and economic impact. Targeting control measures at this stage has shown that a high percentage of cysticercosis cases may be prevented [1,2]. However, such a strategy would be challenging due to lack of well-performing, field-deployable, easy-to-use diagnostic tests to identify infected people. Therefore, there is a need for a validated test that can be deployed in endemic communities.

The existing reference tests for *T. solium* taeniosis are imperfect [3]. *T. solium* taeniosis is diagnosed by self-detection, parasitological, immunological, or molecular methods [4,5]. Parasitological methods are based on morphological identification of eggs, scolices and proglottids from stool samples. Stool microscopy using the Kato Katz thick smear and the formol-ether concentration techniques are routine methods applied to individuals and in epidemiological studies. However, despite a high specificity for *Taenia* species (on genus level), the sensitivity is low, ranging from 38% to 69% [4]. The low sensitivity is due to the nature of the test itself, and because microscopy targets *T. solium* eggs, which are shed irregularly, and not before the end of the pre-patent period. Even when shed, they are not uniformly distributed in the stool. When eggs are available in the sample, it is also not possible to differentiate *Taenia* species based on egg morphology. Other parasitological diagnostic methods are based on the recovery of gravid proglottids and scolices from stool samples. Recovery of parasite material, especially the scolex is generally very difficult. When recovered, the *T. solium* scolex is identified by its double row of hooks on the rostellum, while the proglottids are identified by the three-lobed ovary, absence of vaginal sphincter and especially by the number of uterine branches which are 7–16 compared to *T. saginata*’s 14–32 and *T. asiatica* 12–26 [6,7]. The common immunological method applied on stool samples is the copro antigen ELISA (Copro Ag ELISA) in its various forms. It has been utilized in many epidemiological studies and has a reported sensitivity of 84.5% in an ELISA format [3], which is two and half times better than coproparasitological methods [8]. The initially developed copro Ag ELISA using polyclonal antibodies produced against crude adult worm somatic antigens, yields many false positive results [8,9] and is *Taenia* genus specific. A hybrid, species-specific ELISA utilizing polyclonal antibodies against adult worm somatic antigens and conjugated polyclonal antibodies targeting excretory-secretory antigens has been developed with estimated 96.4% sensitivity and 100% specificity, based on a small sample size [10]. Recently, another species-specific ELISA which utilizes concanavalin A, a lectin, for capturing the antigen and a conjugated monoclonal antibody against adult worm soluble extracts for detection has been developed. It is yet to be fully validated [9]. Several molecular tests have been developed for taeniosis diagnosis on stool samples [11]. Among the commonly used are the nested PCR targeting the Tsol 31 gene (sensitivity 97%, specificity 100%) [12] and the multiplex PCR targeting the cytochrome *c* oxidase subunit 1 gene (detection rate around 50%) [13]. The easiest molecular test developed is the loop mediated isothermal amplification test (LAMP) with an analytical sensitivity of 88% [14]. However, while most molecular tests have a high analytical sensitivity, their performance is limited due to the inconsistent presence of parasite material in the stool sample, especially tapeworm eggs, the source of the nucleic acid. For serum samples, an antibody detecting recombinant protein-based immunoblot test, the rES33 enzyme-linked immunoelectrotransfer blot (rES33 EITB) has been developed with 98% sensitivity and 97% specificity [15]. The parasitological tests perform sub-optimally while the immunological and molecular tests require expensive equipment to be used in laboratory settings by qualified staff. Therefore, these tests cannot be deployed in areas where they are needed most, i.e., community and health posts [16].

There remains a need for a validated rapid, easy-to-use test for taeniosis. Diagnostic test evaluation should be done for the function for which the test will be utilized and in a population as close as possible to the one in which the test will be deployed. Given the differences in the target analytes and test formats of existing reference tests, different results may be obtained when the various tests are applied to the same sample, complicating result interpretation and evaluation of new tests, especially in the absence of a gold standard. In the latter case, it has been suggested that other methods of result analysis such as discrepancy analysis, latent class analysis, composite reference standard, consensus by a panel of experts, test positivity rate or test concordance can be used [17].

Recently, a new two-strip prototype *T. solium* point-of-care test (TS POC) was developed by the Centers for Disease Control and Prevention (CDC), Atlanta, Georgia, USA, in collaboration with the Technical University of Munich (TUM), Munich, Germany, for simultaneous detection of antibodies against the tapeworm (TS POC T) and its intermediate cysticercus stage (TS POC CC) in humans [18]. Epidemiologically, the purpose of the TS POC T is to identify spots of active human transmission while clinically is to identify infected individuals who need to be treated.

The TS POC performance was evaluated through a multi-country study project entitled “Evaluation of an antibody detecting point-of-care test for the diagnosis of *Taenia solium* taeniosis and (neuro) cysticercosis in Tanzania and Zambia” (SOLID). In this study, we report on the diagnostic accuracy outcomes, sensitivity, specificity and predictive values of the community-based TS POC T evaluation and challenges encountered.

## 2. Methods

The methods are detailed in Van Damme and Trevisan et al. [18] and summarized below.

### 2.1. Study Design

**Figure 1:**
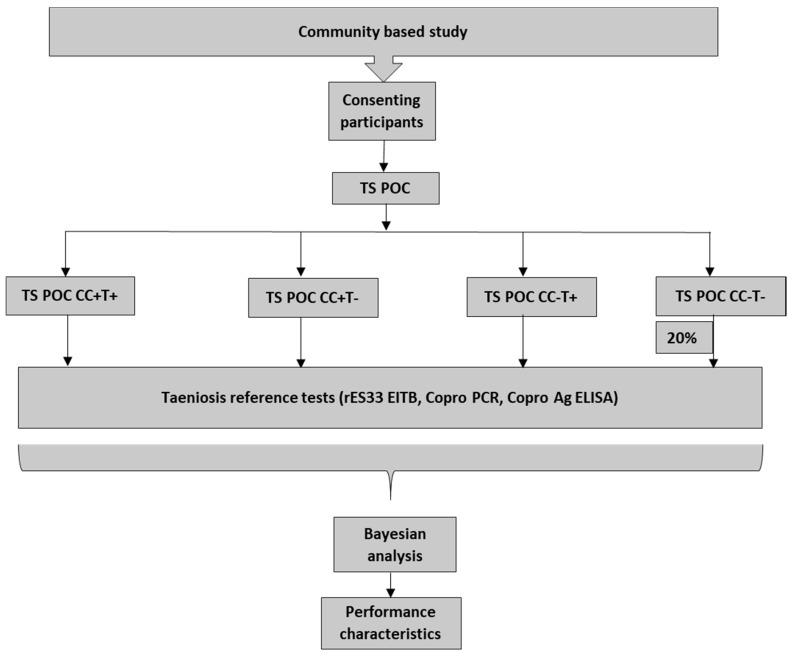
Study design flow.

This prospective diagnostic accuracy study was performed in rural communities in Zambia. Participants were recruited from randomly selected households and were enrolled before obtaining any information on index test or reference tests. All tests were applied to the same study subject. The study had a two-stage design, in which all participants were tested using the TS POC test, after which a subset was tested using the different reference tests. All participants with a TS POC T and/or TS POC CC positive result and a randomly selected 20% subset of participants with a negative result for both test strips, were requested to give a blood and stool sample for reference testing (Figure 1).

### 2.2. Study Area

**Figure 2:**
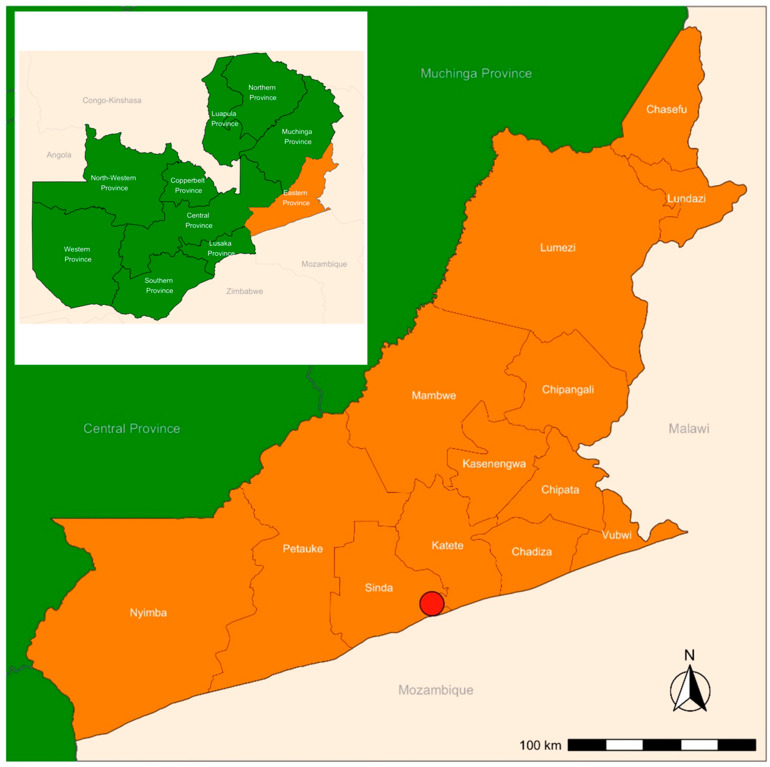
Study area, Sinda district (maroon circle), Eastern province, Zambia.

The study area was Sinda district in the Eastern province of Zambia (Figure 2), selected due to its *T. solium* endemic status, presence of free-roaming pigs and low sanitation levels [19]. Four communities in the catchment of Mtandaza local clinic were selected based on willingness to participate, proximity to the local health center, year round accessibility and reports of porcine cysticercosis [19].

### 2.3. Recruitment of Participants

A pre-study census was carried out in the participating communities. The calculated target sample size of 1200 participants (to obtain a desired precision of 10% [18]) was proportionately allocated to a village based on census results. In each village, the selection of participating households was random. In participating households, every consenting eligible participant was recruited. To be eligible, participants had to be living in the area, be more than 10 years old and express willingness to participate in all study aspects, such as getting tested, and provision of blood and stool samples. Participants who were pregnant, had severe health conditions (self-reported) or were only visiting the area were not eligible for the study. Recruitment continued through several households until the village target number was met. Participant recruitment was conducted from December 2017 to June 2019.

### 2.4. Sampling and Sample Processing

Sampling for the running of reference tests was based on the TS POC results. There was no interval elapsing between TS POC testing and collection of samples for reference testing and no clinical interventions were done between the two tests. All participants who tested positive for taeniosis and/or cysticercosis, and 20% of systematically selected double negatives were asked to give a blood and stool sample. Blood was sampled by a trained nurse or clinician. Three milliliters (3 mL) of venous blood was collected by venipuncture from the arm into plain tubes. Stool samples were collected by giving participants stool pots to go and fill when defecating. The stool pots were returned by the participants either the same day or the following morning.

Blood in plain tubes was put to stand at 4 °C overnight. Samples were centrifuged at 3000 rpm, and the serum was aliquoted into two cryovials to be stored at −20 °C until reference testing. Stool was aliquoted into two 15 mL tubes. One aliquot was put in 10% formalin for copro Ag ELISA, the other in 70% ethanol for copro PCR. They were stored at room temperature until reference testing.

### 2.5. Test Methods

#### 2.5.1. The Prototype *Taenia solium* Point of Care Test (TS POC)

**Figure 3:**
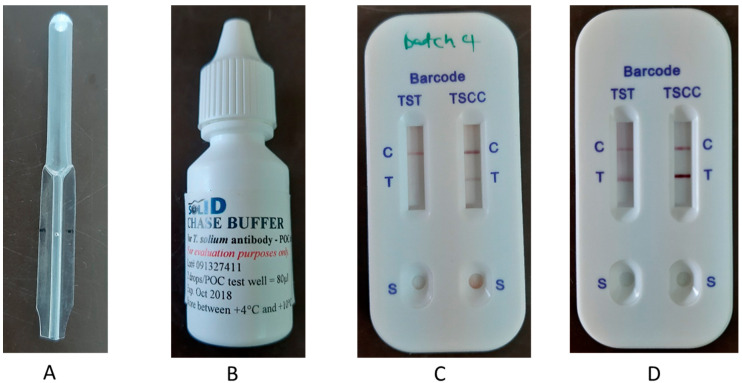
(**A**) Micropipette, (**B**) Chase buffer, (**C**,**D**), TS POC cassettes with (**C**) taeniosis (TST) negative and cysticercosis (TSCC) positive and (**D**) double positive.

The TS POC is an antibody detecting prototype test based on two previously characterized and extensively used recombinant proteins, rES33 and rT24H [20,21]. It is an in-house produced standard lateral flow assay (LFA) which consists of a double-strip cassette (Figure 3) that holds two separate strips: one strip for the detection of taeniosis antibodies (by using the rES33 protein) and one for the detection of cysticercosis antibodies (by using the rT24H protein). Each LFA strip has one test line and one control line and a separate port for the sample application (Figure 3). TS POC cassettes were sealed separately in an aluminum pouch together with a desiccant. Each complete POC kit was labeled with a batch number, production and expiry dates. They were stored between 4–30 °C until use. Preliminary laboratory performance for the TS POC using serum bank sera of confirmed taeniosis (positive microscopic coprology results with PCR confirmation) patients (128), as well as negative control sera and sera from patients with other parasitic infections, such as *Schistosoma haematobium*, *Echinococcus granulosus* (128) was as follows; cysticercosis, sensitivity was 88–93% and specificity 99%; taeniosis, sensitivity was 82% and specificity 99% (CDC, TUM, unpublished).

To perform the test, a finger was disinfected with an ethanol swab, then pricked using a lancet. Whole blood (20 µL) was collected using a micropipette (Figure 3A) and placed in the sample well for taeniosis. The procedure was repeated for cysticercosis using a different micropipette. Two drops of chase buffer (80 µL) were then applied to each well. The timer was started when the sample started running and the results read after 20 min. A positive result was indicated by appearance of a pink line on the test line level and absence of the same line was a negative result. A valid test was one with a pink line on the control level. The results were read by two readers (first a community health worker, followed by a clinician) and if they disagreed, a third reader (often a researcher) was available to break the tie. The readings were recorded on the result card and entered in EpiCollect 5 (https://five.epicollect.net/ last accessed on 28 August 2019) by the researcher.

#### 2.5.2. Reference Tests

Three reference tests were used for the evaluation of the performance of TS POC T for taeniosis detection; rES33 EITB (sensitivity 99%, specificity 99.7%) with a few modifications, the conjugate was used at a dilution of 1:1000 while the 3, 3′-Diaminobenzidine (DAB, 10mg Sigma Aldrich) and the hydrogen peroxide was used according to manufacturer’s instructions [15,22]; copro multiplex PCR for *T. solium* (copro PCR) (sensitivity 67%, specificity, not reported) [13] and copro Ag ELISA (sensitivity 85%, specificity 92%) [23], as modified [24]. DNA for the PCR was extracted using the Qiamp DNA kit according to manufacturer’s instructions.

The reference tests were conducted at the Regional Reference Laboratory in Zambia (copro Ag ELISA) and at the Institute of Tropical Medicine of Antwerp (ITM), Belgium (rES33 EITB, copro PCR). One researcher (CM), who was part of the field recruitment also participated in analysis with the copro Ag ELISA (partially blinded), the other laboratory staff was completely blinded to the TS POC test result as well as the other reference test results. Participant clinical information was not shared to any of the people involved in testing.

### 2.6. Treatment and Purging of Taeniosis Positive Participants

Participants who tested positive for taeniosis on the TS POC T and/or on any reference tests were treated with niclosamide 2 g, single dose and two hours later purged with 30 g of magnesium sulfate. They were given a dish in which to defecate overnight and the following day, the stool was checked for the presence of proglottids. These stool samples were also aliquoted in formalin and ethanol as described above. Species identification was confirmed by nested PCR Restricted Fragment Length Polymorphism (PCR-RFLP) on parasite DNA extracts [25,26]. After reference testing, taeniosis positive participants (that were TS POC T negative) were followed up, treated and purged for tapeworm recovery.

### 2.7. Data Analysis

Sensitivity and specificity of the TS POC T test strip and of the reference tests were estimated using a Bayesian approach with probabilistic constraints as previously described [27,28], but expanding the original approach by altering the multinomial probabilities according to the observed sampling frequencies to incorporate the TS POC T based sampling. Since sampling depended also on TS POC CC results, this was incorporated in a secondary analysis, but no reasonable fits between data and priors were obtained. Open BUGS version 3.2.3 (www.openbugs.net accessed on 15 March 2019) was used. Probabilistic constraints used were in form of expert opinion obtained from three experts from the Institute of Tropical Medicine (PD), Ghent University (SG) and the University of Zambia (KEM) (File S1). Based on these prior estimates, several models were constructed with increasing level of constraints (File S1). Fifty thousand iterations were run and 25,000 discarded as burn-in. Convergence was checked and model fits were compared using the Deviance Information Criterion (DIC), Bayesian P and pD (evaluated in both the multinomial probabilities and the model parameters [28]). Bayesian analysis was applied only to participants who had a valid TS POC T result as well as all three reference tests (complete cases). Indeterminate TS POC T test results arose when the repeat of an invalid test was also invalid. Participants for whom the result was indeterminate were not sampled, and the results were therefore not included in the analysis. For reference tests, no indeterminate results were recorded.

Besides the sensitivity and specificity, the positive/negative predictive values of the TS POC T test and of the three reference tests, and the prevalence of taeniosis, were estimated using the Bayesian model. For each of the different test combinations, Cohen’s kappa point estimate was calculated, weighing the observed frequencies according to the proportion of complete cases within each TS POC result combination. Since positive and negative agreements are more informative for rare findings, these indexes were also calculated, according to Cicchetti and Feinstein [29] using the weighted frequencies to account for the proportion of complete cases.

### 2.8. Trial Registration

Pan African Clinical Trials Registry identifier: PACTR201712002788898. Accessed on 21 November 2017, https://pactr.samrc.ac.za/TrialDisplay.aspx?TrialID=2788.

## 3. Results

### 3.1. Flow and Demographic Characteristics of Participants

**Figure 4:**
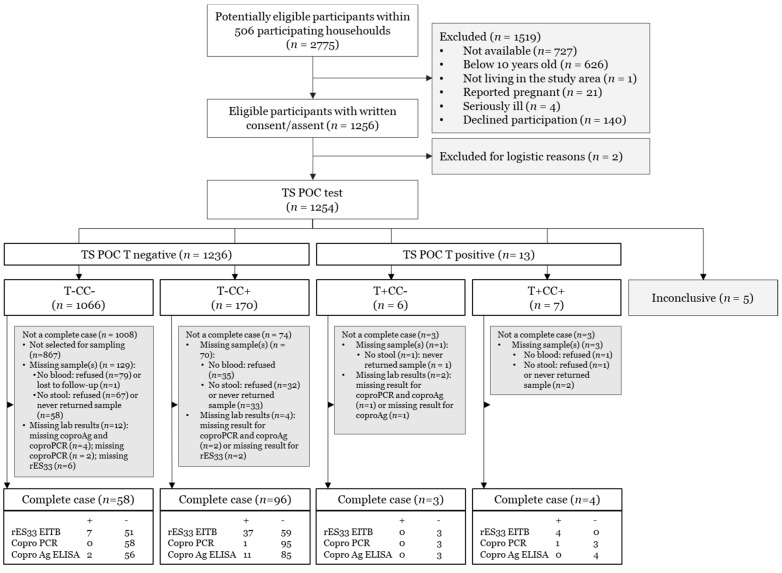
Flow diagram showing community recruitment, TS POC T and reference testing.

From the pre-study census, the four communities consisted of 39 different villages with 862 households and a total population of 4331 people. Within 506 participating households, 2775 participants were screened for eligibility. From the screened participants, 1256 were eligible, and 1254 were tested with the TS POC test (Figure 4). Among the tested participants, 536 (43%) were male and 718 (57%) were female. The mean age was 33, ranging between 10 and 95 years.

Out of the 1254 participants who were tested with the TS POC, 13 tested positive for taeniosis (Figure 4). From these, 12 serum and 9 stool samples were collected. From the 1236 participants who tested negative with the TS POC T test, 1066 tested negative using both the TS POC T and TS POC CC test strip. Out of the 199 (1066-867) participants that were selected for sampling in this group, 119 serum and 74 stool samples were obtained. Out of the 170 participants who tested TS POC T negative and TS POC CC positive, 135 serum and 105 stool samples were collected. No adverse event was recorded when performing the TS POC T or any of the reference tests. Five participants had an inconclusive TS POC result and were not requested to give a sample. Overall, a sample, either serum, stool, or both, was obtained from 273 participants. In total, 266 serum and 188 stool samples were collected. A total of 179 participants provided both a blood and stool sample. Out of these participants, 18 had one or more missing reference test results (Figure 4), resulting in a total of 161 complete cases that were used for the primary analysis. The result combinations of the TS POC T and the reference tests of the complete cases is given in Table 1.

### 3.2. Bayesian Estimates of Performance Characteristics

Based on the prior information, several models were built, ranging from very loose to very strict priors (File S1). For the primary analysis, model 2B was selected based on DIC, Bayesian P and pD. The models using more confided priors, resulted in bad fits as expressed in Bayesian P-values close to 1. The best performing model had a Bayesian P-value of 0.53, the lowest DIC and the best correspondence between pD based on the multinomial probabilities and pD based on the model parameters (5.4 vs. 4.4). Although the latter values indicate that priors were not restrictive enough, this model was considered the best to fit the data at hand. The Bayesian estimates of sensitivity, specificity, positive and negative predictive values of the TS POC T, rES33 EITB, copro PCR and copro Ag ELISA are given in Table 2. The estimated sensitivity and specificity for the TS POC T test were 38% (95% CI: 5–93%) and 99% (95% CI: 98–100%), respectively.

### 3.3. Agreement between Tests

Cohen’s kappa estimates were calculated to explore the agreement between each of the tests. As judged by the low kappa estimates, none of the tests showed a substantial agreement (Table 3). Nevertheless, due to the low number of positive tests, low kappa estimates may not accurately reflect the agreement, so positive and negative agreements were also calculated [29]. Although the negative agreements were rather high for all test combinations, the positive agreements were low. None of the complete cases tested positive by all four tests and only few subjects tested positive using two or three of the tests (Table 1). Even within the stool-based tests (copro PCR and copro Ag ELISA), none of the samples tested positive using both methods, resulting in 0% positive agreement (Table 3). Although the TS POC T and rES33 EITB use the same antigen, the positive agreement was only 7%.

### 3.4. Treatment and Purging of Test Positive Participants

Each of the 13 participants who tested positive using the TS POC T test during recruitment was treated and purged. Proglottids were collected from one person and the sample tested positive for *T. solium* using PCR-RFLP (copro Ag ELISA was negative). Additionally, 71 out of 74 participants who tested negative using the TS POC T, but tested positive using any one of the reference tests, were followed up in the community for treatment and purging. Sixty-nine (69) were treated and purged. Out of these, 58 gave a stool sample after being purged. From these samples, no worm, proglottid or scolex was recovered. The analysis of the stool samples using the reference tests and the organization of field visits to seek out positive participants inherently resulted in a time lag of at least one month between the collection of the primary sample and treatment/purging. Therefore, the results after treatment/purging of reference positive participants should be interpreted with caution.

## 4. Discussion

The aim of this study was to assess the sensitivity and specificity of the TS POC T test for the detection of taeniosis in resource-poor, *T. solium* endemic communities in sub-Saharan Africa. The estimated sensitivity of the TS POC T (38%, 95% CI 5–93%) was imprecise, but still lower than the 95% threshold defined in the stool-based target product profiles for *T. solium* taeniosis diagnosis [30]. Therefore, the sensitivity is too low, even for a rural community where affordability and access are considered more important than accuracy [11]. On the other hand, the estimated specificity of the TS POC T was high, and was estimated with a high precision (99%, 95% CI 98–100%). Of course, only 13 participants (out of 1249 participants with a valid result) tested positive using the TS POC T, which means that the number of false positive cases could not be higher than 13. When treated and purged, proglottids could only be collected and confirmed in one of the 13 TS POC T positive participants. Although a high specificity becomes more important for control programs when the prevalence decreases [31], the low sensitivity makes the test in its current form unfit for purpose. At this level of performance, it is recommended that the sensitivity of the TS POC T test be increased. This can be attempted by optimizing the concentration of the reagents in the LFA, including the antigen. However, given the low level of sensitivity of the current test and the discordance with the rES33 EITB that uses the same antigen, the format of the test may need to be redesigned.

Sensitivities and specificities of the reference tests were also estimated during this study. A sensitivity of 98% had previously been reported for the rES33 EITB (on field reference samples) [15], which is a similar sensitivity to what we found (95%). The high estimated sensitivity may have been induced by the high prior information used in the model. In contrast, the specificity of the rES33 EITB was lower (74%, CI 70–79%) than the 99% that was previously reported [15]. In the latter study, only sera from non-endemic countries were used. In our study conducted in a highly endemic area, the possibility of past taeniosis infections/exposure cannot be excluded. Moreover, the relatively low specificity could also be due to possible cross reactions with *Schistosoma*, *Ascaris* and *Plasmodium* exposed people or exposure to antigens which are similarly expressed in the metacestode and adult stages of the parasite [15,32,33]. Indeed, a lot of samples were included in the analysis based on cysticercosis positivity on the other strip (TS POC CC). A previous study has shown high positivity (46%) of the rES33 EITB on cysticercus positive samples [15], which was also evident in our study. Unfortunately, our analysis was not able to account for the impact of the TS POC CC result on the sampling since there was no agreement between data and priors when the TS POC CC was added to the analysis. Since TS POC CC positive participants are overrepresented in our sample, this may have caused biased estimates.

The sensitivities of the stool-based reference tests were lower than previously reported. One study had reported the sensitivity and specificity of the copro Ag ELISA at 85% and 92%, respectively [3]. The copro PCR was previously reported with a positive detection rate of nearly 70% when compared to copro-parasitological methods [34]. There could be several reasons for the low point estimates of the sensitivities for the copro PCR and copro Ag ELISA. One reason could be that the stool-based reference tests target present infections, but they have been evaluated in the present study in combination with tests that also target exposure. Moreover, the use of uninformed prior information is known to affect posterior outcomes in a Bayesian analysis [27], but the use of weaker priors in our final model was necessitated by the low agreement between prior information and the data. While this achieved model convergence and moderate agreement between data and the model, it resulted in wide credibility intervals. Another reason for the wide credibility intervals of the sensitivities may be related to the low number of true positive taeniosis cases in our sample. This is related to the low taeniosis prevalence, although the prevalence of 1.8% (95% CI 0.2–4.5) from this study is similar to the 0.6% point prevalence previously reported in the same area using stool-based tests [3]. For low prevalence diseases, the number of participants without the disease (which are necessary to determine the specificity) will be adequate to obtain sufficiently narrow confidence/credibility intervals for the specificity. Therefore, we used a two-stage design, in which only a subset of the test negatives was sampled to reduce the excessive number of reference tests in true negative cases. Nevertheless, since this selection was based on the TS POC test (which had a lower point estimate for sensitivity than initially anticipated), we may have excluded some true positive cases (which are required to estimate the sensitivity). Besides, also due to the high refusal rate, particularly for stool samples, the number of true positive cases may have been reduced, since cases with a missing reference test result were excluded from the primary analysis. Besides the lower sample size, this may also reduce generalizability of the study when the disease spectrum among the refusing population would be different from the participating population. Therefore, future studies should take these limitations into account when estimating the sensitivity of a taeniosis test with a higher level of precision.

The cross tabulation of results showed a large variation in the number of positive cases each test detected and low agreement among them (discordance). This has also been recorded in previous studies [3,9,24,35,36,37,38,39]. In addition, it has been reported that no single taeniosis diagnostic technique will “always” detect infection [37,40,41]. Nevertheless, discordance among reference tests in our study was striking since none of the samples was positive for all the reference tests. Six samples were positive on at least two reference tests. Similar to our results, high differences in the number of positive cases detected and discordance between tests were observed in nearly all studies where taeniosis reference tests have been applied to field samples [3,24,35,36,38,39,42]. High difference in the number of detected cases and discordance among taeniosis reference tests presented challenges in our evaluation study such that, the methods of handling imperfect tests [17] may produce sub-optimal performance estimates. The discordance in test results may be reduced by the use of reference tests targeting the same analyte as the index test, such as antibody, antigen or DNA, in the same matrix (stool, blood or urine) [43]. Nevertheless, for taeniosis, this option is not available, as so far there is only one antibody detecting test available for serum (rES33 EITB) [15,44].

With the currently available diagnostic tools for taeniosis, a multi-testing system might need to be considered for a more accurate detection of cases whereby a case definition can be used to increase sensitivity or specificity depending on the testing purpose. An investigation into cost and epidemiological/clinical implications of a trade-off between sensitivity and specificity may need to be done. Given the many existing stool-based reference tests, there is need to investigate the combination of tests that give the best diagnostic performance.

## 5. Conclusions

The lack of a gold standard remains the principal challenge in the evaluation of taeniosis diagnostic tests. Using currently available *T. solium* taeniosis reference tests presents challenges such as high variability in the number of positive cases detected and test discordance. As exemplified during the evaluation of the TS POC T, this cascades into challenges in estimating performance characteristics. Regarding the performance of the TS POC T, optimizing the antigen concentration and of other reagents in the test could potentially improve sensitivity (while keeping specificity sufficiently high) to a sufficient threshold to allow utilization of the test for screening in endemic areas such as Zambia.

## Figures and Tables

**Table 1:** Result combination between the TS POC T and reference tests for complete cases (*n* = 161).

TS POC T	rES33 EITB	Copro PCR	Copro Ag ELISA	*n*
1	1	1	1	0
1	1	1	0	1
1	1	0	1	0
1	1	0	0	3
1	0	1	1	0
1	0	1	0	0
1	0	0	1	0
1	0	0	0	3
0	1	1	1	0
0	1	1	0	1
0	1	0	1	4
0	1	0	0	39
0	0	1	1	0
0	0	1	0	0
0	0	0	1	9
0	0	0	0	101

TS POC T = *T. solium* point of care test for taeniosis; rES33 EITB = recombinant antigen 33kDa enzyme-linked immunoelectrotransfer blot; copro PCR = copro multiplex PCR for *T. solium;* copro Ag ELISA = copro antigen enzyme-linked immunosorbent assay Complete cases who are TS POC T positive (*n* = 7) are sampled from 13 TS POC T positive participants, and the TS POC T negative complete cases (*n* = 154) are from 1236 TS POC T negative participants. NOTE: 1 = positive, 0 = negative.

**Table 2:** Performance characteristics of the TS POC T and the reference tests.

Parameters	TS POC T	rES33 EITB	Copro PCR	Copro Ag ELISA
Se (95% CI)	38 (5–93)	95 (91–98)	42 (6–93)	40 (12–74)
Sp (95% CI)	99 (98–100)	74 (70–79)	99 (97–100)	91 (85–95)
PPV (95% CI)	40 (5–80)	6 (1–15)	45 (4–96)	7 (1–25)
NPV (95% CI)	99 (96–100)	100 (99–100)	99 (96–100)	99 (97–100)

Bayessp = 0.53 (0–1); Prevalence 1.8% (0.2–4.5%); CI = Credibility interval; Se = sensitivity; Sp = specificity; PPV = positive predictive value; NPV = negative predictive value; TS POC T = *T. solium* point of care test for taeniosis; rES33 EITB = recombinant antigen 33kDa enzyme-linked immunoelectrotransfer blot; copro PCR = copro multiplex PCR for *T. solium;* copro Ag ELISA = copro antigen enzyme-linked immunosorbent assay. Priors: prevalence 0.1–5%; rES33 EITB: sensitivity 90–99% and specificity 70–99%; all other tests: sensitivity 0–100% and specificity 50–100%.

**Table 3:** Agreement between different tests for the detection of taeniosis.

	TS POC T	rES33 EITB	Copro PCR
TS POC T	NA ^a^		
rES33 EITB	κ = 0.05 * p_pos_* = 0.07 * p_neg_* = 0.91	NA	
Copro PCR	κ = 0.21 * p_pos_* = 0.21 * p_neg_* = 0.99	κ = 0.03 * p_pos_* = 0.03 * p_neg_* = 0.91	NA
Copro Ag ELISA	κ = −0.02 * p_pos_* = 0 * p_neg_* = 0.97	κ = −0.02 * p_pos_* = 0.06 * p_neg_* = 0.89	κ = −0.01 * p_pos_* = 0 * p_neg_* = 0.98

Cohen’s Kappa test statistic (κ), positive agreement (*p_pos_*) and negative agreement (*p_neg_*) were calculated based on the results of the complete cases (*n* = 161), after inverse weighting the observed frequencies to account for the sampling design and missingness, assuming missing at random. ^a^ NA, not applicable.

## Data Availability

Data is readily available at the Institute of Tropical Medicine, Antwerp, through the Data Access Committee: https://www.itg.be/E/data-sharing-open-access (accessed on 5 October 2021), email: ITMresearchdataaccess@itg.be. However, it cannot be shared publicly due to ethical and privacy concerns.

## Supplementary Materials

The following are available online at https://www.mdpi.com/article/10.3390/diagnostics11112039/s1, File S1. Prior information, models and model results. File S2. Study protocol. File S3. STARD checklist.
