# Supplementary material for: Challenges Encountered When Evaluating an Antibody-Detecting Point-of-Care Test for Taeniosis in an Endemic Community in Zambia: A Prospective Diagnostic Accuracy Study
Source: Diagnostics (Basel). 2021 Nov 4;11(11):2039. doi: 10.3390/diagnostics11112039 (PMC8625551; doi:10.3390/diagnostics11112039)
Supplement: Supplementary file 1 [file diagnostics-11-02039-s001.zip › diagnostics-1431851-supplementary/TS POC T supplementaryfiles/File S1_Expert prior information and models 26092021.pdf]

File S1

Expert prior information

Probabilistic constraints used were in form of expert opinion obtained from three experts from the Institute of Tropical Medicine (PD), Ghent University (SG) and the University of Zambia (KEM). The analysis allows the sensitivities and specificities to differ conditional on the other test-results

Expert priors on prevalence, sensitivity and specificity.

Parameter	Lower limit	Upper limit
Prevalence	0.001	0.05
rES33 EITB – sensitivity	0.9	0.99
rES33 EITB – specificity	0.7	0.99
Copro mPCR Tsol sensitivity	0.7	0.99
Copro mPCR Tsol specificity	0.9	0.99
Copro Ag ELISA sensitivity	0.9	0.99
Copro Ag ELISA specificity	0.3	0.8

Models

Models used for POC-T analysis

Parameters	Model1	Model2	Model2B	Model3	Model4
theta1	[0;0.05]	[0;0.05]	[0;0.05]	[0;0.05]	[0;0.05]
theta2	[0;1]	[0;1]	[0;1]	[0;1]	[0;1]
theta3	[0.5,1]	[0.5,1]	[0.5,1]	[0.5,1]	[0.5,1]
theta4	[0;1]	[0.9;0.99]	[0.9;0.99]	[0.9;0.99]	[0.9;0.99]
theta5	[0;1]	[0.9;0.99]	[0.9;0.99]	[0.9;0.99]	[0.9;0.99]
theta6	[0.5,1]	[0.7;0.99]	[0.7;0.99]	[0.7;0.99]	[0.7;0.99]
theta7	[0.5,1]	[0.7;0.99]	[0.7;0.99]	[0.7;0.99]	[0.7;0.99]
theta8	[0;1]	[0;1]	[0;1]	[0.7;0.99]	[0.7;0.99]
theta9	[0;1]	[0;1]	theta8	[0.7;0.99]	[0.7;0.99]
theta10	[0;1]	[0;1]	theta8	[0.7;0.99]	[0.7;0.99]
theta11	[0;1]	[0;1]	theta8	[0.7;0.99]	[0.7;0.99]
theta12	[0.5,1]	[0.5,1]	[0.5;1]	[0.9;0.99]	[0.9;0.99]
theta13	[0.5,1]	[0.5,1]	theta12	[0.9;0.99]	[0.9;0.99]
theta14	[0.5,1]	[0.5,1]	theta12	[0.9;0.99]	[0.9;0.99]
theta15	[0.5,1]	[0.5,1]	theta12	[0.9;0.99]	[0.9;0.99]
theta16	[0;1]	[0;1]	[0;1]	[0;1]	[0.9;0.99]
theta17	[0;1]	[0;1]	[0;1]	[0;1]	[0.3;0.8]

theta18	[0;1]	[0;1]	[0;1]	[0;1]	[0.9;0.99]
theta19	[0;1]	[0;1]	[0;1]	[0;1]	[0.3;0.8]
theta20	[0;1]	[0;1]	[0;1]	[0;1]	[0.9;0.99]
theta21	[0;1]	[0;1]	[0;1]	[0;1]	[0.3;0.8]
theta22	[0;1]	[0;1]	[0;1]	[0;1]	[0.9;0.99]
theta23	[0;1]	[0;1]	[0;1]	[0;1]	[0.3;0.8]
theta24	[0.5,1]	[0.5,1]	[0.5,1]	[0.5,1]	[0.9;0.99]
theta25	[0.5,1]	[0.5,1]	[0.5,1]	[0.5,1]	[0.3;0.8]
theta26	[0.5,1]	[0.5,1]	[0.5,1]	[0.5,1]	[0.9;0.99]
theta27	[0.5,1]	[0.5,1]	[0.5,1]	[0.5,1]	[0.3;0.8]
theta28	[0.5,1]	[0.5,1]	[0.5,1]	[0.5,1]	[0.9;0.99]
theta29	[0.5,1]	[0.5,1]	[0.5,1]	[0.5,1]	[0.3;0.8]
theta30	[0.5,1]	[0.5,1]	[0.5,1]	[0.5,1]	[0.9;0.99]
theta31	[0.5,1]	[0.5,1]	[0.5,1]	[0.5,1]	[0.3;0.8]
