# Supplementary material for: Challenges Encountered When Evaluating an Antibody-Detecting Point-of-Care Test for Taeniosis in an Endemic Community in Zambia: A Prospective Diagnostic Accuracy Study
Source: Diagnostics (Basel). 2021 Nov 4;11(11):2039. doi: 10.3390/diagnostics11112039 (PMC8625551; doi:10.3390/diagnostics11112039)
Supplement: Supplementary file 1 [file diagnostics-11-02039-s001.zip › diagnostics-1431851-supplementary/TS POC T supplementaryfiles/File S2_Protocol_SOLID_Z_V1.3_29082017.pdf]

Evaluation of an antibody detecting point-of-care test for the diagnosis of *Taenia solium* taeniosis and (neuro)cysticercosis in community settings of highly endemic, resource-poor areas in Zambia

Protocol SOLID Z Version 1.3, dated 29-08-2017

*

**Sponsor/Commissioner:**	Institute of Tropical Medicine<br>Nationalestraat 155<br>B-2000 Antwerp - Belgium
**Coordinating Investigator:**	E. Bottieau, Department of Clinical Sciences, ITM, Belgium
**Study Coordinator**	S. Gabriël, Ghent University, Belgium
**Principal Investigator**<br>**Co- Principal Investigators**	G. Zulu, Ministry of Health, Zambia<br>C. Chabala, School of Medicine of the University of Zambia, Zambia<br>K.E. Mwape, University of Zambia

**Protocol Number:**	**Protocol SOLID Z Version 1.3, dated 29-08-2017**
**Title:**	Evaluation of an antibody detecting point-of-care test for the diagnosis of *Taenia solium* taeniosis and (neuro)cysticercosis in community settings of highly endemic, resource-poor areas in Zambia
**Version:**	**Version 1.3**
**Partnering institutions:**	- *Belgium*: Institute of Tropical Medicine (ITM), Antwerp (Coordination) (P. Dorny, E. Bottieau, C. Trevisan); Ghent University, Faculty of Veterinary Medicine (S. Gabriël, I. Van Damme), Faculty of Sciences (E. Abatih)<br>- *Germany*: Technical University of Munich, Department of Neurology (V. Schmidt, A. Winkler)<br>- *Denmark:* University of Copenhagen, Faculty of Health and Medical Sciences, (M. V. Johansen, P. Magnussen, K. Schou Moller [PhD student]); Statens Serum Institut (R. Stensvold)<br>- *Tanzania:* National Institute for Medical Research, Muhimbili Medical Research Centre (B. Ngowi); Ministry of Health (B. Ndawi); Sokoine University of Agriculture (H. Ngowi)

	- *Zambia:* University of Zambia, School of Veterinary Medicine (K. E. Mwape, I.K. Phiri; C. Mubanga [PhD student]); University of Zambia , School of Medicine (C. Chabala); University Teaching Hospital (O. Ciccone), Ministry of Health (G. Zulu)

**Funder:**	EDCTP (European and Developing Countries Clinical Trials Partnership), BMBF (German Federal Ministry of Education and Research)
**Sponsor:**	Institute of Tropical Medicine, Antwerp, Belgium
**Coordinating Investigator:**	E. Bottieau
**Department:**	Department of Clinical Sciences
**Address:**	Institute of Tropical Medicine, Nationalestraat 155, 2000 Antwerp, Belgium
**Telephone:**	+32 3 247 64 50
**Fax:**	
**Email:**	ebottieau@itg.be

TABLE OF CONTENTS

Statement of Compliance & Confidentiality 6
LIST OF ABBREVIATIONS 7
Synopsis 8
1.0 Introduction* 9
1.1 Background and rationale* 9
1.2 The project SOLID 10
1.3 Impact of expected results 13
1.4 Main objective 13
1.5 Specific objectives: 14
2.0 Literature review 14
2.1 Taeniasis 15
2.1.1 Microscopy 15
2.1.2 rES33 15
2.1.3 Copro Antigen ELISA 15
2.1.4 Copro PCR 16
2.1.5 Loop mediated Isothermal Amplification Test 17
2.2 Cysticercosis and Neurocysticercosis 17
2.2.1 Antigen ELISA 17
2.2.2 Antibody ELISA 17
2.2.3 rT24 Immunoblot test (Recombinant Enzyme linked Immuno Electro blot (EITB)) 18
2.2.4 Lentil lectin Glycoprotein Enzyme Linked Immuno-electrotransfer blot (LLGP EITB) 18
2.2.5 Neuroimaging (Computed Tomography scan and Magnetic Resonance Imaging (MRI)) 19
2.2.6 Neurocysticercosis diagnostic criteria 19
3.0 Methods 21
3.1 Study Design 21
3.2 Study Setting, Population and Sampling Strategy 21
3.2.1 Sample Size and Power 23
3.2.2 Inclusion and Exclusion Criteria 24
3.3 Procedures 25
3.3.1 Data Collection 25
3.3.2 Participant related Interventions and Procedures 27
3.3.3 Laboratory Procedures 32
3.4 Data Analysis 36
3.5 Data Management and Archiving 37
3.5.1 Data Management 37
3.5.2 Archiving 38
3.5.3 Open access to research data 38
3.6 Dissemination of results 39
3.7 Ethical Issues 39
3.7.1 Ethical (and Regulatory) Review 39
3.7.2 Obtaining Informed Consent 41
3.7.3 Insurance 42
3.7.4 Obligations of investigators 42
3.7.5 Benefits, harms and risks of study participation 43
3.8. Monitoring and Quality Control 45
4.0 Budget 47

5.0 Project timelines......48

6.0 References......50

7.0 Appendices......54
- **7.1 Information sheet in English** 54
- **7.2 Information sheet in Chewa** 58
- **7.3 Informed consent form in English** 63
- **7.4 Informed consent form in Chewa** 66
- **7.5 SOLID partners** 69
- **7.6 Detailed sample size calculations** 70

INSTITUTE OF TROPICAL MEDICINE ANTWERP

STATEMENT OF COMPLIANCE & CONFIDENTIALITY

The information contained in this study protocol is privileged and confidential. As such, it may not be disclosed unless specific permission is given in writing by the ITM or when such disclosure is required by federal or other laws or regulations. These restrictions on disclosure will apply equally to all future information supplied which is privileged or confidential.

Once the final protocol has been issued and signed by the Investigator(s) and the authorized signatories, it cannot be informally altered. Protocol amendments have the same legal status and must pass through the mandatory steps of review and approval before being implemented.

By signing this document, the Investigator commits to carry out the study in compliance with the protocol, the applicable ethical guidelines like the Declaration of Helsinki and consistent with international scientific standards as well as all applicable regulatory requirements. The Investigator will also make every reasonable effort to complete the study within the timelines designated.

PRINCIPAL INVESTIGATOR:*

Title, Name: DR GIDEON ZULU Date: 08/09/2017

Signed: [signature]

CO - PRINCIPAL INVESTIGATOR:*

Title, Name: Date:

Signed:

CO - PRINCIPAL INVESTIGATOR:*

Title, Name: Date:

Signed:

COORDINATING INVESTIGATORS (IF APPLICABLE):*

Title, Name: Date:

Signed:

STATEMENT OF COMPLIANCE & CONFIDENTIALITY

The information contained in this study protocol is privileged and confidential. As such, it may not be disclosed unless specific permission is given in writing by the ITM or when such disclosure is required by federal or other laws or regulations. These restrictions on disclosure will apply equally to all future information supplied which is privileged or confidential.

Once the final protocol has been issued and signed by the Investigator(s) and the authorized signatories, it cannot be informally altered. Protocol amendments have the same legal status and must pass through the mandatory steps of review and approval before being implemented.

By signing this document, the Investigator commits to carry out the study in compliance with the protocol, the applicable ethical guidelines like the Declaration of Helsinki and consistent with international scientific standards as well as all applicable regulatory requirements. The Investigator will also make every reasonable effort to complete the study within the timelines designated.

PRINCIPAL INVESTIGATOR:*

Title, Name: Date:

Signed:

CO - PRINCIPAL INVESTIGATOR:*

Title, Name: Dr Chishala Chabala Date: 11th September 2017

Signed:

CO - PRINCIPAL INVESTIGATOR:*

Title, Name: Date:

Signed:

COORDINATING INVESTIGATORS (IF APPLICABLE):*

Title, Name: Date:

Signed:

STATEMENT OF COMPLIANCE & CONFIDENTIALITY

The information contained in this study protocol is privileged and confidential. As such, it may not be disclosed unless specific permission is given in writing by the ITM or when such disclosure is required by federal or other laws or regulations. These restrictions on disclosure will apply equally to all future information supplied which is privileged or confidential.

Once the final protocol has been issued and signed by the Investigator(s) and the authorized signatories, it cannot be informally altered. Protocol amendments have the same legal status and must pass through the mandatory steps of review and approval before being implemented.

By signing this document, the Investigator commits to carry out the study in compliance with the protocol, the applicable ethical guidelines like the Declaration of Helsinki and consistent with international scientific standards as well as all applicable regulatory requirements. The Investigator will also make every reasonable effort to complete the study within the timelines designated.

PRINCIPAL INVESTIGATOR:*

Title, Name: Date:

Signed:

CO - PRINCIPAL INVESTIGATOR:*

Title, Name: Date:

Signed:

CO - PRINCIPAL INVESTIGATOR:*

Title, Name: Dr. Kabemba Evans Mwape Date: 11/09/2017

Signed:

COORDINATING INVESTIGATORS (IF APPLICABLE):*

Title, Name: Date:

Signed:

STATEMENT OF COMPLIANCE & CONFIDENTIALITY

The information contained in this study protocol is privileged and confidential. As such, it may not be disclosed unless specific permission is given in writing by the ITM or when such disclosure is required by federal or other laws or regulations. These restrictions on disclosure will apply equally to all future information supplied which is privileged or confidential.

Once the final protocol has been issued and signed by the Investigator(s) and the authorized signatories, it cannot be informally altered. Protocol amendments have the same legal status and must pass through the mandatory steps of review and approval before being implemented.

By signing this document, the Investigator commits to carry out the study in compliance with the protocol, the applicable ethical guidelines like the Declaration of Helsinki and consistent with international scientific standards as well as all applicable regulatory requirements. The Investigator will also make every reasonable effort to complete the study within the timelines designated.

PRINCIPAL INVESTIGATOR:*

Title, Name: ______________________ Date: ____________

Signed:

CO - PRINCIPAL INVESTIGATOR:*

Title, Name: ______________________ Date: ____________

Signed:

CO - PRINCIPAL INVESTIGATOR:*

Title, Name: ______________________ Date: ____________

Signed:

COORDINATING INVESTIGATORS (IF APPLICABLE):*

Title, Name: Pr Dr BOTTIEAU EMMANUEL Date: 11/09/2017

Signed:

LIST OF ABBREVIATIONS

Ab	Antibody
Ag	Antigen
CC	Cysticercosis
CDC	Centers for Disease Control and Prevention
CRO	Contract Research Organization
CT	Computed Tomography
DB	Database
ELISA	Enzyme-Linked Immunosorbent Assay
GCP	Good Clinical Practice
GEP	Good Epidemiological Practice
GLP	Good Laboratory Practice
IC(F)	Informed Consent (Form)
(I)EC	(Independent) Ethics Committee
IRB	Institutional Review Board
ITM	Institute of Tropical Medicine
LLGP-EITB	Lentil-Lectin Purified Glycoprotein Enzyme-Linked Immunoelectrotransfer Blot
NCC	Neurocysticercosis
PI	Principal Investigator
POC	Point-of-care
QC	Quality Control
RRL	Regional Reference Laboratory
(S)AE	(Serious)Adverse Event
SDV	Source Data Verification
SOP	Standard Operating Procedure
STARD	Standards for Reporting Diagnostic accuracy studies
T	Taeniosis
WPs	Work Packages

SYNOPSIS

Taenia solium taeniosis/cysticercosis (T/CC) is a neglected zoonotic parasitic disease complex with significant economic and public health impacts. Neurocysticercosis (NCC) is estimated to be responsible for 30% of cases of acquired epilepsy in endemic areas. Currently, there are no cheap, easy to apply, sensitive and specific diagnostic tools available for the detection of this parasite. The lateral flow test from Centers for Disease Control and Prevention, Atlanta that combines the diagnosis of (N)CC with the diagnosis of taeniosis, is a very promising candidate. The main objective of the SOLID project is to contribute to the implementation of a rapid, cheap and simple point-of-care (POC) test (= index test) for the detection of *T. solium* taeniosis (T) and (N)CC in two resource-poor, highly endemic areas in sub-Saharan Africa (Tanzania and Zambia), including capacity building.

This protocol covers the POC test diagnostic accuracy studies at **community level** in Zambia. More specifically the objectives are to evaluate the diagnostic accuracy of the POC test for NCC and for T in randomly selected study participants living in the community.

Based on the sampling design of this study, households will be randomly sampled and all consenting eligible study participants within the selected households will be included and tested with the POC test. The POC test combines two well-validated recombinant antigens Ag – rT24H (detection of **CC-Antibodies (Ab))** and rES33 (detection of **T-Ab**) – in one test, study participants will result in POC positive and POC negative, both for CC and T, leading to 4 possible combinations. Depending on the POC test results a blood and stool sample for reference testing will be collected. Furthermore, all POC CC positives and a random subset of POC CC negatives will undergo an additional neurological examination performed by the study neurologist and be invited for a CT scan if necessary.

All patients positive for T will be treated with niclosamide or if not available, praziquantel. A specific patient management and follow up of close contacts will be put in place. All symptomatic patients will be invited to visit the rural health centre.

A Bayesian approach will be implemented to evaluate the performance of the POC test with respect to reference tests, clinical and CT scan data. The main outcome will be the POC test performance though the estimation of its sensitivity and specificity.

1.0 INTRODUCTION*

1.1 Background and rationale*

Taenia solium taeniasis/(neuro)cysticercosis (T/(N)CC) is a neglected zoonotic parasitic infection/disease complex ranked first on the global scale of foodborne parasites by FAO/WHO (FAO/WHO, 2014), and the need for control/elimination was recognized by the World Health Assembly (WHO, 2013). Humans can be carrier of the tapeworm in the intestines (taeniosis, T), but can also act as an accidental dead-end intermediate host (besides the normal pig intermediate host). The metacestode larval stage can develop in the muscles or subcutaneous tissue (cysticercosis, CC), but often also enters the central nervous system where neurocysticercosis (NCC) subsequently develops. NCC is estimated to be responsible for 30% of cases of acquired epilepsy in endemic areas (Ndimubanzi et al. 2010) and its contribution to chronic daily headache is assumed to be substantial although epidemiological data is lacking. Knowledge regarding the distribution of T/(N)CC is very scarce in sub-Saharan Africa (Braae et al., 2015). For Zambia, high prevalences of *T. solium* have been described, more specifically, in the Eastern Province of Zambia a seroprevalence of human CC of 33.5-38.5% (antibody detection) and of T of 6.3-11.9% was determined, ranking among the highest in the world (Mwape et al. 2012; Mwape et al. 2013) . Another study in the same region indicated over 50% of the acquired epilepsy cases to be due to NCC (Mwape et al. 2015).

A recent WHO expert consultation identified the urgent need for field applicable, accurate diagnostic tools for T/(N)CC, which are currently not available (WHO, 2014) and to our knowledge, no field evaluation studies on new diagnostic tools are on-going or planned. Diagnosis and management of NCC cases is mainly based on a combination of neuroimaging, assisted by serology (lentil lectin-bound glycoproteins - enzyme-linked immunoelectrotransfer blot, (LLGP-EITB)) (there is no gold standard test). In resource poor areas, this is problematic as access to these neuroimaging tools is very limited or non-existent, and the LLGP-EITB, though widely recognised with a high specificity (Sp) and sensitivity (Se) (Tsang et al. 1989), is expensive and in a format not applicable/available in most resource poor laboratories.

Affordable and accurate point-of-care (POC) diagnostics are as such urgently required. So far, one POC assay for detection of CC in serum using a recombinant antigen (Ag) – rT24H - has been developed by Corstjen at al. (2014) in collaboration with the Centers for Disease Control and Prevention (CDC), Atlanta, and performed very well (Corstjen et al., 2014). Clinical Se and Sp of this assay to detect cases of CC with two or more viable brain cysts were 96% and

98%, respectively. Unfortunately, this lateral flow test still required a portable lightweight strip analyser, which limits the use in African field settings. Therefore, a modified lateral flow assay, which can be read visually, has now been developed by CDC Atlanta combining two well-validated recombinant Ag – rT24H (detection of **CC-Ab)** and rES33 (detection of **T-Ab**) – in one test kit. The rT24H represents a recombinant protein, which is based on the well-known specific 8-kD glycoprotein fraction of the LLGP-EITB (current accepted gold standard for CC-Ab detection) and was evaluated in an immunoblot format, with a Se of up to 99% and Sp of 100% (Noh et al., 2014). Recombinant and synthetic antigens including the rt24H Ag were tested in an immunoblot format on a large number of negative and confirmed CC positive samples and compared with the LLGP-EITB. Concordance between the recombinant EITB and the LLGP-EITB was almost perfect (kappa = 0.89), with the rt24H Ag performing best. Field validation of the rt24H antigen blot test has been extensively done, mainly in Peru in collaboration with CDC, Atlanta confirming its excellent performances. This work is yet to be published. The rES33 is based on an excretory-secretory protein of the adult tapeworm (Levine et al. 2004), and was previously thoroughly evaluated in an immunoblot format, on a large panel of serum samples from endemic (Peru) and non-endemic areas (US, Egypt), demonstrating its high performance in terms of sensitivity and specificity (Sensitivity of 99% and a specificity of 99.7%). The successful use of rES33 in a lateral flow format has been described in the paper of Handali et al., (2010)(Handali et al. 2010). Both recombinant antigens already proved to be highly Se and Sp in different assay formats and were chosen for the present POC assay to facilitate a future standardized low-cost production of this test for resource poor settings. This POC test allows the simultaneous detection of CC (by rT24H) and T (by rES33) with only 10µl serum or 20 µl whole blood per sample port obtained by finger prick (diagnosis based on specific Ab detection). Large-scale field evaluation of this test is required.

1.2 The project SOLID

The proposed project 'SOLID', consists of three work packages (WPs) (Figure 1), all three WP will be conducted as a collaborative effort involving partners from Belgium, Denmark, Germany, Tanzania and Zambia. The total duration of the SOLID project is four years, with an expected completion date in August 2020.

The POC test will be evaluated for the diagnosis of NCC, CC and T at community level in Zambia and at rural district hospital (DH) level in Tanzania. **WP1**, entails the evaluation of the

POC test, and is subdivided into five sub WPs. In WP1.1 field/hospital based studies will be conducted where participants/patients will be enrolled and samples needed to evaluate the POC test collected. According to a predefined flow, a number of participants/patients will receive a CT scan for NCC diagnosis according to predefined criteria. In all cases of test positivity (any test), specific patient follow up will be put in place, including the close contacts of the patients and CT scans where clinically required. The CT scans and patient management will be covered by WP1.2. WP1.3 will ensure the delivery of the POC test (= index test) on the study sites and laboratories. WP1.4 covers the analyses of the collected samples with the reference tests for T and CC at the regional reference laboratory (RRL), with subsets tested in Belgium and Denmark for quality control. This will lead to data needed for the assessment of the POC test performance for T/(N)CC at community and DH level. WP1.5 will perform data management and analyses.

WP2 is responsible for the organisation of all capacity building, including technology transfer and training. This will be done working to the principles of good clinical practice (GCP) and good clinical laboratory practices (GCLP). All WPs work closely together and will be coordinated by **WP3**. The latter WP also includes the dissemination and contributions to the endorsement/implementation process.

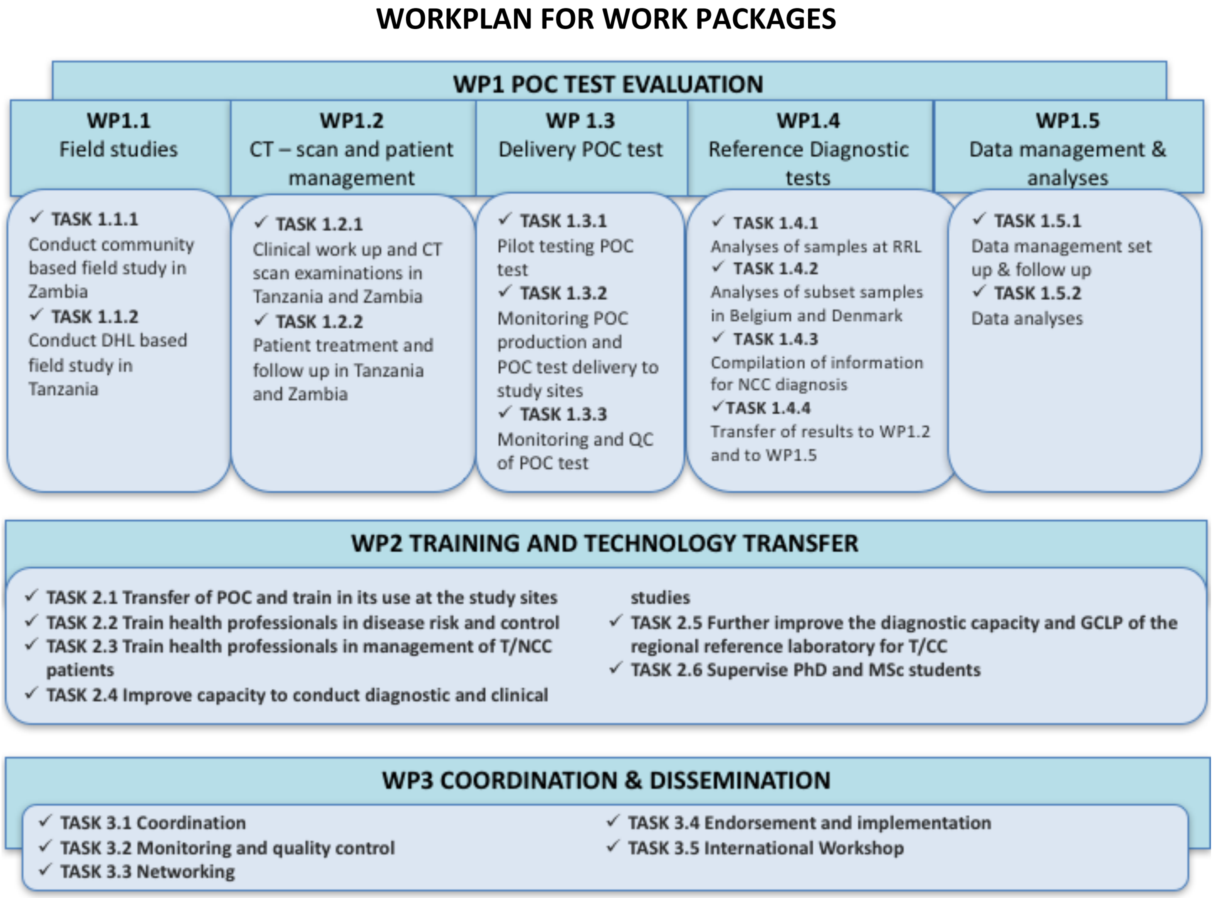

Figure 1: Work plan for work packages (WPs), activities and tasks. CC: Cysticercosis; CT: Computed Tomography; GCLP: Good clinical laboratory practices; NCC: Neurocysticercosis; POC: Point-of-care; QC: Quality control; RRL: Regional reference lab; T: Taeniosis.

The SOLID project will have PhD and MSc students all with their own objectives. These will be defined when the students start to prepare their own protocol. Amendments to this protocol will be requested if required.

The consortium of project proposers consists of medical doctors with experience in the field of *T. solium*, neurologists, parasitologists and statisticians (See Table in Appendix 7.5): a multidisciplinary team following the One Health approach with a lot of experience in capacity building and large-scale observational and interventional studies in developing countries on *T. solium* and other parasitic zoonoses. The Regional Reference Laboratory in Zambia is a project partner as well.

1.3 Impact of expected results

If the proposed test is successfully validated, WHO is fully prepared to endorse it, and it can be proposed for implementation.

The impact of the implementation/availability of the POC test will be visible on multiple levels:

1. The tool will be the first diagnostic test for (N)CC available at DH level. Combined with training of the staff, this will enable the DH to correctly and early diagnose suspected NCC cases and initiate referral of patients to neuroimaging facilities. This will lead to improved patient management practices, which will have a positive effect on their health outcome. Additionally, a correct diagnosis and knowledge of NCC will potentially stimulate the people's perception of epilepsy as being a biological disease (e.g. NCC as its cause) rather than a consequence of witchcraft). Creating awareness and educating professionals in disease recognition would further lead to an increased number of detected cases.
2. The tool will be the first POC test for T available at DH level. This will lead to an early detection of tapeworm carriers. The latter is crucial to reduce/stop disease transmission as tapeworm carriers are responsible for the environmental contamination with infective eggs. These carriers represent a risk not only for themselves, but also for their close contacts. Early detection will ensure a rapid halt to this environmental contamination reducing the risk of new NCC cases and of infection of pigs and thus of perpetuating the life cycle. The training will guide the staff in treating and following up on not only the tapeworm carrier but also the patients' household contacts for suspected NCC.
3. The implementation of the tool planned at the level of the community health workers (village level), which will enable an early detection of T cases and suspected NCC cases, which can subsequently be referred to the DH where a decision on further diagnostic work-up will be made. This will not only impact positively on patients' health outcome, but also on the community, as disease transmission will be blocked at an early stage, avoiding new cases and infection of pigs. The community will also be informed/sensitized on this new diagnostic test.

1.4 Main objective

The **main objective** of the SOLID project is to contribute to the implementation of a rapid, cheap and simple POC test for the detection of *T. solium* T and (N)CC in a resource poor, highly endemic areas in Tanzania and Zambia, including capacity building.

The proposed study protocol will evaluate the diagnostic accuracy of the POC test, which simultaneously detects (N)CC and *T. solium* T at **community level** in Zambia.

1.5 Specific objectives:

- To evaluate the diagnostic accuracy of the POC test for (N)CC in randomly selected study participants living in the community
- To evaluate the diagnostic accuracy of the POC test for T in randomly selected study participants living in the community

2.0 LITERATURE REVIEW

Diagnosis and case management of Neurocysticercosis in endemic countries is problematic. This is due to the lack of diagnostic tools with sufficient performance and in formats that are cost effective, easy to use and suitable for large scale implementation in resource limited settings (WHO, 2015). T(N)CC is increasingly becoming an important disease globally, especially so with the immigrant pressure on developed countries (Nash *et al.*, 2015). Therefore, there is increasing urgency not only to control but eradicate the disease in endemic areas. Mass interventions as required for disease control and eradication are dependent on accurate and reliable diagnostic tests. Currently, the existing tests are imaging tests, Magnetic Resonance Imaging (MRI) , Computed Tomography (CT) scan, histopathology and generally tests that provide immunological evidence of contact between hosts and parasites (Del Brutto *et al.*, 2017a) These tests are expensive and limited. Some tests like MRI are largely restricted to research and not readily available for diagnostic purposes for the public. All the tests available for NCC require sophisticated infrastructure and trained personnel to run and manage them. Besides these general limitations, individual tests have specific limitations inherent to them (Lightowlers *et al.*, 2016a). As such, recognition has arisen that in order to establish baselines, measure intervention impacts, undertake regular surveillance or establish clinical diagnoses, ready to use and inexpensive diagnostic tests are required. These tests should also be able to accurately diagnose NCC cases in community settings. To date, there remains a need to develop the accurate and reliable diagnostic test that will meet the draft target profile that was set by the WHO panel of experts on Taeniasis/ Neurocysticercosis (WHO, 2015).

A number of diagnostic tests already exist. However, each test has inherent characteristics that limits its use in a full scale intervention. The following are the common diagnostic tests in use today for taeniasis, cysticercosis and neurocysticercosis.

2.1 Taeniasis

2.1.1 Microscopy

Microscopy has been used to identify *T. solium* as well as to distinguish it from *T. saginata* (E.M. Elsdon-Dew, 1965). Both coprology as well as histology has been used. The main features visualized are the scolex, the proglottids and the eggs. However, the sensitivity and the specificity of microscopy are low. The scolices are not easy to obtain and when obtained they may be damaged morphologically to be able to identify differential features. Mature gravid proglottids are usually the only material available. Identification is usually based on the number of uterine branches as well as the "accessory third lobe" to the uterus. The eggs for the two worms cannot be differentiated on microscopy. The eggs are also only shed intermittently (Soulsby, 1982; Mayta *et al.*, 2000).

2.1.2 rES33

Wilkins et al (1999) developed an immunoblot assay for the detection of Taeniasis using Taenia Solium Excretory Secretory antigen (TSES). This test had a sensitivity of 95% and specificity of 100%. However, there were problems with this test. Firstly, the crude antigen was being produced from infecting immunosuppressed hamsters. Thus, the purification process was labor intensive, time consuming and costly. For example, the turnaround time was three months and the production of antigen from each batch was very limited. The complexity of crude TSES made it not be used in other test formats (Levine *et al.*, 2004). In order to overcome these short comings, the secretory excretory proteins were characterized, cloned and expressed in baculovirus to produce two immunogenic recombinant antigens rES33 and rES38. These antigens were tested on an immunoblot test (EITB) and were found sensitive for Taeniasis. They did not cross react with Cysticercosis serum, thus specific (Levine *et al.*, 2004). Despite this breakthrough, this test suffers from the same limitations of being an antibody detecting test as discussed below in 2.4.1.3.

2.1.3 Copro Antigen ELISA

A Copro antigen ELISA developed by Allan et al existed (Allan and Craig, 2006) with modifications by Mwape (Mwape *et al.*, 2012). This test though was only genus specific, therefore, could not discriminate between *T. saginata* and *T asiatica*. Based on this problem, another Copro antigen ELISA was developed by using the antibodies against the genus specific

antigen and conjugated IgG antibodies against the Excretory-Secretory *T solium* specific protein. This test had a sensitivity of 96.4% and 100% specificity with no cross reactions with other helminthes (Guezala *et al.*, 2009). The limitation of this test is similar to that of all ELISAs in that they are unable to differentiate between active and inactive infections, as well as require expensive laboratory infrastructure and highly trained technical staff. Beyond this, the source of the antigens is crude extracts whose use presents difficulties as discussed with the EITB above.

2.1.4 Copro PCR

Several Copro PCRs have been developed. Some have achieved high sensitivity as well as specificity. However, generally, PCR is technically demanding and very expensive. Isolation of DNA from fecal samples is laborious due to the presence of PCR inhibitory substances (Mathis and Deplazes, 2006). As such, its application to monitor a large scale intervention programme is limited. PCR though is suitable for confirmative purposes or identification of taeniid eggs recovered from the environment or from fecal samples. The following are some of the Copro PCR that a WHO panel of experts recognized;

- Tsol31 Nested Copro PCR

  This PCR was developed to overcome some observed bottle necks in previous PCR tests. It has been developed to use stool as a DNA source. Its sensitivity was reported to be 96.9% while specificity was 100%. It had a detection limit of 10 eggs/250mg fecal sample (Mayta *et al.*, 2008).
- Multiplex PCR

  This Multiplex PCR was developed for differential diagnosis of Taeniid species especially in co-infections. However, this test still requires field validation as it was tested on samples as old as 12 years old(Yamasaki *et al.*, 2004). Other researchers evaluated this test in comparison with other tests gave a sensitivity of 37.2%, which is very low (Nkouawa *et al.*, 2009).
- Real time Copro PCR

  This real time PCR has been developed and tested on field samples. Using a Bayesian modeling, its test characteristics were compared with coprology and Copro antigen ELISA. The real time PCR had sensitivity and specificity of 82.7% and 99%, respectively (Praet *et al.*, 2013a).

2.1.5 Loop mediated Isothermal Amplification Test

This test was developed to overcome some of the short comings of PCR, Copro tests and serology (Nkouawa *et al.*, 2009). The test uses stool samples. Its sensitivity was determined to be 88.4%. The test time was 90 minutes which is way faster than PCR. Compared to PCR, it is relatively cheaper and requires less equipment. However, it still requires a water bath, which makes it not suitable for field application (Nkouawa *et al.*, 2010).

2.2 Cysticercosis and Neurocysticercosis

2.2.1 Antigen ELISA

This test detects circulating secretory/excretory antigens from viable or living cysticerci. There have been many serological tests developed to detect circulating antigens. However, two tests have been frequently evaluated (Rodriguez, Wilkins and Dorny, 2012) and the WHO recognizes these two antigen ELISA tests (WHO, 2015); (1) The ELISA test based on the HP10 monoclonal antibody (Harrison *et al.*, 1989) and (2) the sandwich antigen ELISA test based on the B158/B60 monoclonal antibodies (Dorny *et al.*, 2000). Both tests were originally developed for cysticerci of *Taenia saginata* but cross reacted with cysticerci of *T. solium* in both human and pig samples, hence the adoption. They also cross reacts with *T. hydatigena* (Rodriguez, Wilkins and Dorny, 2012). In view of this, their specificity needs to be interpreted carefully (Lightowlers *et al.*, 2016b). The sensitivity of both tests is good when there are more than two viable cysts (Rodriguez, Wilkins and Dorny, 2012). The B158/B60 has a sensitivity of 84.5% and a specificity of 99% (Praet *et al.*, 2013b).

These tests being ELISA, they also require laboratory infrastructure and skilled personnel. The B158/B60 Ag ELISA has already been commercialized (apDIA, Belgium). It costs €145 per kit translating to €3.15 per sample which is significantly high if scaled up (Lightowlers *et al.*, 2016b).

2.2.2 Antibody ELISA

The WHO recognizes two antibody detecting ELISAs; (1) the one based on oncosphere antigens (Ferrer *et al.*, 2005) and (2) the one based on the crude antigen extract (Diaz *et al.*, 1992). Ferrer et al (2005) evaluates the performance of five synthetic peptides in an antibody ELISA. The best results obtained were on a combination of three peptides giving a sensitivity

of 85% and specificity of 83.5%. The test using individual peptides produced varied results. The antibody ELISA by Diaz et al had Sensitivity of 65% and Specificity of 63% on serum samples. On CSF, the sensitivity was 62%, the specificity was not reported. The ELISA also cross reacted with *Echnococus granulosus* and *Hymenolepis nana* (Diaz *et al.*, 1992). Both tests have limitations for field utilization inherent to all ELISAs.

2.2.3 rT24 Immunoblot test (Recombinant Enzyme linked Immuno Electro blot (EITB))

This test is also an antibody detecting test based on the same principles as the LLGP EITB but uses recombinant antigen rT24 in an immunoblot test. The T24 was one of the seven antigen proteins that was used in the LLGP EITB test that was successfully recombined and cloned to rT24. It has been tested in an immunoblot test and its sensitivity and specificity has been found to be similar to the LLGP EITB (Hancock *et al.*, 2006; Noh *et al.*, 2014). Based on this recombinant antigen, a lateral flow test has been developed and evaluated with a sensitivity and specificty of 96% and 98% respectiviely. While this is a major breakthrough, the test comes with a lightweight strip analyser which makes it not so portable (Corstjens *et al.*, 2014). This is the same antigen that has been used in the Point of Care (POC) lateral flow test which this study is evaluating.

2.2.4 Lentil lectin Glycoprotein Enzyme Linked Immuno-electrotransfer blot (LLGP EITB)

This is an antibody detecting test. It uses seven antigenic glycoproteins that are purified from a metacestode homogenate by lentil lectin chromatography. Its initial sensitivity and specificity were reported at 98% and 100% respectively (Tsang, Brand and Boyer, 1989). This is a test of choice and one of the reference antibody tests (Rodriguez, Wilkins and Dorny, 2012). Despite its high sensitivity and specificity, this test has its short comings. In its original form, the source of the antigen is fresh cysts from pigs. Thus purification of the antigen is dependent on having a source of parasites, and requires expensive sophisticated equipment and expertise. The purification process for LLGP antigens from cysts has also been difficult to standardize (Hancock *et al.*, 2006; Noh *et al.*, 2014). LLGP EITB has a complicated procedure, not quantitative (Rodriguez, Wilkins and Dorny, 2012) and requires good laboratory infrastructure, therefore, not suitable for field use. The western blot based test is reportedly very expensive (Lightowlers *et al.*, 2016b). Beyond this, being an antibody detecting test, it is unable to discriminate between active and inactive infections. Therefore, it cannot differentiate between exposure and active infection.

2.2.5 Neuroimaging (Computed Tomography scan and Magnetic Resonance Imaging (MRI))

These imaging techniques not only improved NCC diagnosis but also replaced the old radiography based techniques to diagnose NCC. Imaging allows the; determination of the location of the cysts whether neural or not; topography of the lesions; stage of involution and degree of inflammation. They also allow the identification of the presence of the scolex which is nearly considered pathognomonic of NCC (Del Brutto *et al.*, 2017b). Despite these advantages, this imaging equipment especially MRI is expensive and scarcely found in resource poor endemic countries (Garcia and Del Brutto, 2003). Thus, these diagnostic tools if present in endemic countries often only exist for research purposes or at national referral centers. For example, in Zambia, only one MRI unit was available in 2015 and exclusively used for research purposes only (WHO, 2015). The CT scans are also limited to urban hospitals. In times of scaled up interventions, resources may not permit the use of such expensive diagnostic tools except in special cases to monitor the outcomes of the interventions (Lightowlers *et al.*, 2016b)

2.2.6 Neurocysticercosis diagnostic criteria

Diagnosis of NCC has been based on criteria developed and periodically upgraded by Del Brutto (Del Brutto *et al.*, 2017a). Modifications to this criteria has also enhanced the definitive diagnosis especially in endemic areas where imaging equipment is limited (Gabriel *et al.*, 2012). This criteria is based on probability of diagnostic certainty of particular tests which are combined to produce two diagnostic outcomes, definitive diagnosis and probable diagnosis. These criteria are as follows;

2.4.2.1 Absolute criteria

This criteria provides absolute evidence of Cysticerci infection of the central nervous system. Three sub-criteria constitutes the absolute diagnostic criteria; (1) histological demonstration of the parasite from biopsy of brain or spinal cord tissue, (2) visualization of sub-retinal cysts and (3) conclusive demonstration of a scolex within a cystic lesion on neuroimaging

2.4.2.2 Neuro imaging criteria

This criteria is subdivided into three sub- criterion, major, confirmative and minor criterion. The major criterion is when the lesions on CT or MRI are suggestive of NCC but cannot be used to confirm or exclude the disease such as lesions with no discernible scolex, enhancing lesions, multi-lobulated cysts in the sub-arachnoid space and typical parenchyma brain calcifications. Confirmative neuro imaging criteria constitutes resolution or transformation of

cysts on follow up imaging either as a result of treatment or spontaneously. The minor criteria is when lesions are compatible with NCC on imaging but are nonspecific as they can also be observed in other diseases, infectious or non-infectious.

2.4.2.3 Clinical or exposure criteria

This provides evidence of possible contact with the parasite. The sub-criteria includes a positive test on the available immunodiagnostic tests, demonstration of cysts outside the CNS, evidence of household contact with T. solium, clinical manifestations supportive of NCC and living or coming out of an endemic area.

2.4.2.4 Degrees of diagnostic certainty

Based on degrees of diagnostic sensitivity, there are two classes of diagnoses, Absolute and probable NCC

Absolute NCC

- Having one absolute criterion
- Having at least two major Neuro imaging criteria plus one exposure criteria
- One major exposure plus one confirmatory exposure and one clinical exposure

Probable NCC

- One major criteria plus two strong exposure criteria
- One minor criteria plus two strong exposure criteria
- Seizures plus two strong exposure criteria

There has been a proposal to include antigen ELISA as a major criteria based on the 2012 diagnostic criteria. This proposal has been evaluated in comparison with the Del Brutto criteria of 2012. Researchers concluded that the two criteria had limitations when used for diagnosis of individual patients though they were useful in epidemiological studies (Del Brutto, 2012; Gabriel *et al.*, 2012; Mwape *et al.*, 2013)

3.0 METHODS

3.1 Study Design

The study is a diagnostic accuracy study according to the Standards for Reporting Diagnostic accuracy studies (STARD) guidelines (STARD, 2015) and will evaluate the performance of POC test, an index test for the combined diagnosis of T and (N)CC, against standard reference tests. Specifically, for Zambia the study will be conducted at community level (Figure 2) in Sinda district of the Eastern province of Zambia, an area highly endemic for *T. solium*.

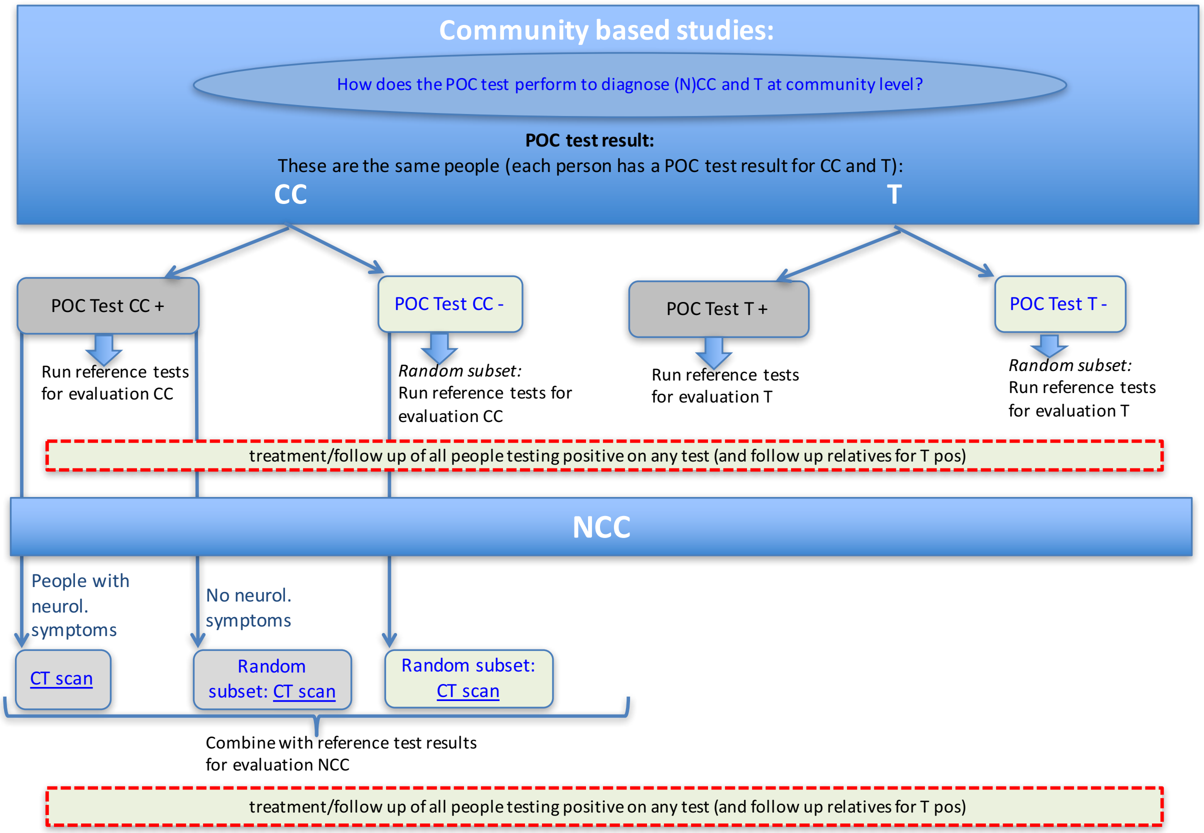

Figure 2: The flow chart showing the study design for the POC test performance at community level in Zambia. CC: Cysticercosis; CT: Computed tomography; NCC: Neurocysticercosis; POC: Point-of-care; T: Taeniosis.

3.2 Study Setting, Population and Sampling Strategy

Study setting

The field studies will be conducted in Sinda district of Eastern Province of Zambia (Figure 3). The study area is well known by the researchers and recognised for its high *T. solium* endemicity, low sanitation levels and mostly free-range pig management.

Suitability of communities in the Sinda district was assessed during a pilot survey conducted by the proposers in April 2015. The eligibility criteria for selection of communities were as follows:

1. Willingness of community to participate
2. Proximity to rural health centre (RHC) and willingness of staff to cooperate
3. Accessible by road all year round, even during the wet season
4. Rural setting with free roaming pigs and reports of porcine CC

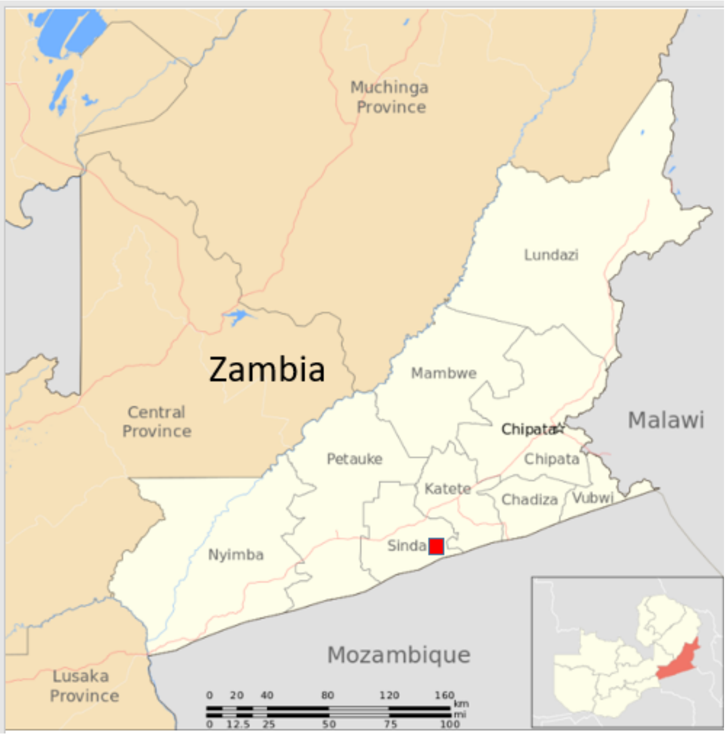

Figure 3: Map of Zambia showing Sinda district and the study site indicated by

Population and Sampling Strategy

In this community based study the villages within the communities and the households within the villages will be selected randomly. All eligible household members consenting to participate will be invited to participate and be tested with the POC test. Prior the sampling, the community will be informed about the project and the activities that will be conducted during a community information day.

3.2.1 Sample Size and Power

The sample size is based on the expected Se and Sp of the POC test for CC and T, a fixed precision of the point estimate of d, and an estimated prevalence of a positive CC or T in the study population of P% (Detailed analysis in Appendix 7.6).

For a single CC cyst a Se of 88% and Sp of 99%, while for 2 or more CC cysts a Se of 93% and Sp of 99% is expected for the POC test, respectively. For T a Se of 82% and Sp of 81% is expected. These test performance results were based on a laboratory evaluation on reference samples performed at CDC, Atlanta.

Based on an Ab detection test (EITB) the prevalence of human CC was reported to be of 33.5-38.5% in Zambia (Mwape et al. 2013). For T, an expected prevalence of 5.9% was assumed for the same study area (unpublished results from the study district).

For the study, two kinds of adjustments are imminent: adjustments based on the sampling design scheme and those based on contingencies during sampling in the field.

Based on the sampling design of this study, households will be randomly sampled and all consenting eligible individuals within the selected households will be included. Given that the number of individuals within households in these communities varies between 2 and up to 20 individuals, a clustering effect is expected. The clustering effect will be adjusted by multiplying the estimated sample size by a value between 1.5 and 2 (Banoo et al., 2010). It often happens that even after sending out notifications prior to visiting households, one or more members of a household are not present during sampling and even after repeated attempts targeted participants cannot be sampled. In order not to reduce the targeted sample size the sample size is increased by between 5% to 20% (Banoo et al., 2010).

Simulations for the sample sizes were computed for varying values of the Se and the expected prevalence of T and CC.

Based on the information provided, the simulations (10,000 runs) indicated that a sample size for T ranging from a minimum of 672 to a maximum of 14,050 study participants and for CC from a minimum of 251 to a maximum of 1,574 study participants is necessary for the study.

As the POC test provides results for both T and CC study participants will be sampled only once for both diseases. Hence, for the community based POC evaluation 1,200 participants will be enrolled. This sample size falls within the ranges of both simulations ensuring a valid evaluation of the test performance and is in accordance with the project budget.

An estimated total of 177 CT scans will be performed including the required scans of household contacts. An estimated 672 samples will be tested with reference tests, of which 444 for CC and 228 for T.

However, a mid-term analysis will be done after 350 samples for CC and at 700 samples for T have been collected, in order to re-estimate the sample size and make adjustments where necessary.

3.2.2 Inclusion and Exclusion Criteria

Inclusion criteria:

In order to be eligible, study participants (or subject groups) **must meet the following criteria:**

Study participants:

- Patients willing and able to participate in all aspects of the study, including providing blood and stool samples, participating in a questionnaire survey, and undergoing brain CT scan if indicated (clinically or per protocol)
- Willing and able to provide written informed consent (assent for minors with consent from a parent or a legally authorised representative)
- Living, attending school, or regularly visiting the bore holes present in the study communities
- Aged 10 years of age or older

Exclusion criteria:

Potential participants (or subject groups) meeting any of the following criteria **will not be enrolled in the study:**

Study participants:

- Unwilling or unable to participate in some or all aspects of the study, including providing blood and stool samples, participating in a questionnaire survey, and undergoing brain CT scan if indicated (clinically or per protocol)
- Unwilling or unable to provide written informed consent (assent for minors with consent from a parent or a legally authorised representative)
- Living outside of, and not regularly visiting, or attending school in, the study communities

- Children below the age of 10 years
- Children below the age of 10 years with severe malnutrition
- Reported pregnant
- Seriously ill (unable to engage in the normal activities of daily living without assistance because of the illnesses)

3.3 Procedures

3.3.1 Data Collection

Figure 4 illustrates the flow chart for the selective study participant enrolment at community level. All household members selected and consenting to participate will be tested with the POC test (Informed consent form in English Appendix 7.3 and Chewa in Appendix 7.4).

This will result in POC positive and POC negative participants, both for CC (POC_CC) and T (POC_T), as the POC test is a combined test.

The work up will depend on the test results (four possible scenarios):

In patients **POC_CC + and T + :** a blood and stool sample will be taken for reference testing (See 3.3.3.2 Other laboratory procedures). T will be treated with niclosamide (drug of choice, single dose - 2g orally) or praziquantel (single dose - 10mg/kg orally) according to drug's availability. The study participant will be send to the primary health facility (rural health centre of the study area) where they will get a neurological examination by the study neurologist. If neurological symptoms are present a CT scan of the brain will be performed (See 3.3.2.2 CT scan, diagnosis of NCC). A 10% randomly selected subset of participants with no neurological symptoms will be forwarded for a CT scan as well (NCC cases can be asymptomatic and symptoms like epileptic seizures and severe headache can take years to develop). The reference laboratory tests results combined with CT scan results will be used for the diagnostic ascertainment of NCC, against which the performance of the POC test will be assessed. Patients diagnosed with NCC will be treated as outlined below (See 3.3.2.3 Patient treatment and follow up). A specific patient management and follow up of close contacts will be put in place.

In patients **POC_CC + and T - :** a blood and stool sample will be taken for reference testing (See 3.3.3.2 Other laboratory procedures). The study participant will be send to the primary health facility where they will get a neurological examination. If neurological symptoms are

present a CT scan of the brain will be performed (See 3.3.2.2 CT scan, diagnosis of NCC); a random 10 % of asymptomatic study participants will also be invited for a brain CT scan. The reference laboratory tests results combined with CT scan results will be used for the diagnostic ascertainment of NCC, against which the performance of the POC test will be assessed. Patients diagnosed with NCC will be treated as outlined below (See 3.3.2.3 Patient treatment and follow up).

In patients **POC_CC - and T + :** a blood and stool sample will be taken for reference testing (See 3.3.3.2 Other laboratory procedures); T will be treated with niclosamide (drug of choice, single dose - 2g orally, drug of choice) or praziquantel (single dose - 10mg/kg orally) according to the drug's availability. A specific patient management and follow up of close contacts will be put in place. A random 10% of asymptomatic study participants will also be invited for a neurological examination and brain CT scan without injection of contrast.

In patients **POC_CC - and T - :** a blood and stool sample will be taken for reference testing (See 3.3.3.2 Other laboratory procedures) in a 20% random subset. A neurological examination and CT scan of the brain (See 3.3.2.2 CT scan, diagnosis of NCC) will be performed at random in 50% of this random subset without injection of contrast (10% of POC_CC – and T-).

In all cases of POC or reference test positivity (for CC and/or T), a specific patient follow up will be put in place, including the close contacts of the study participants and CT scans where clinically required.

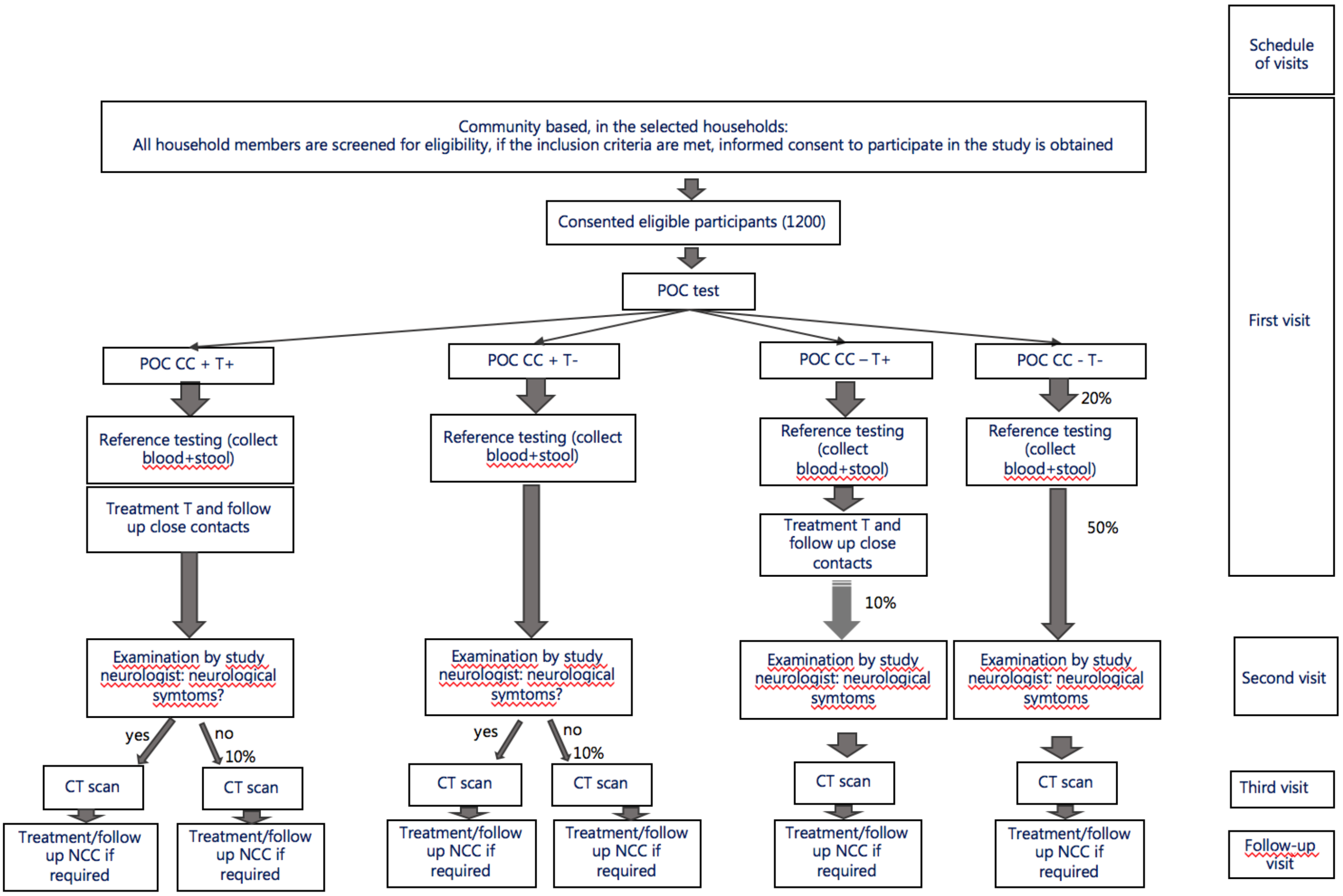

Figure 4: Flow chart for the selective study participant enrolment at community level.CC: cysticercosis; CT: Computed tomography; NCC: Neurocysticercosis; NCZ: Niclosamide; POC: point-of-care; PZQ: Praziquantel; T: taeniosis

3.3.2 Participant related Interventions and Procedures

3.3.2.1 Biomedical data collection

Registration (including informed consent) and biomedical sampling will be conducted by door to door visits of the selected households by suitably qualified and trained personnel with a trained health worker.

POC test

A sample for the POC test (thumb prick) will be obtained as well as the samples for further reference testing if indicated by the protocol (see Figure 4). All collected samples will be

assigned a unique study identification number (code) and labelled using indelible ink or printed stickers with barcodes.

For the performance of the POC test, each device will be labelled with a barcode sticker, clearly linking the device to the participant code. A fingertip of the participant will be disinfected thoroughly with an alcohol swab. Finger prick will be performed using a lancet (e.g. BD micro-fine lancets). Whole blood (20 µl) will be collected and dispensed to each sample port on the device using the disposable micro-pipettor provided. After letting the sample enter the port for a few seconds, 80 µl (about 3 drops) of chasing buffer will be added. After 15 minutes the reading of the test should be performed by two independent readers and in case of discordance, a third reader will be used. In the event of an invalid result (e.g. control line missing), testing needs to be repeated. Results will be entered on forms in addition to a cell phone based database.

Reference sample collection

When reference samples need to be collected, the participant will be requested to provide10mls of a blood sample for further testing in the laboratory and this will be collected by trained medical personnel. The participant will also be provided a stool sample bottle to provide a stool sample for further testing.

All study participants invited to the rural health centre (see Figure 4) will be examined by the study neurologist.

The study doctor will perform a basic neurological examination and mental state work up. Study participant's personal and clinical data will be collected using appropriate case report forms (CRF) according to principles of GCP. Depending on the neurological examination result and according to the flow chart participants will be offered a CT scan. These however will take place on a later stage. During the waiting period, symptomatic patients will be managed and treated according to the mental health/epilepsy patient management practices (see section 3.3.2.3 Patient treatment and follow up). Patients with chronic progressive headache will be instructed to visit the rural health centre if other symptoms arise.

3.3.2.2 CT scan, patient management:

After clinical evaluation, including history taking and examination for potential contra-indications, a CT scan of the brain with and without contrast (if there is no clinical contra-

indication) will be performed in consenting patients, for all POC CC test positives and a subset of the POC CC test negatives (see Figure 4). The patients will receive full information of the nature of the study, involved investigations and implications of a positive outcome. A trained radiologist will counsel the patient during the procedure and perform the imaging CT scanning will be done at Chipata Central Hospital in Chipata or at Levy Mwanawasa General Hospital in Lusaka, depending on CT scan availability. Interpretation of the CT scans results will be discussed between a German neurology post-doc, local radiologist and the senior neuroradiologist from the Technical University of Munich, Germany. Inter-rater agreement will be assessed.

CT scans will be evaluated according to the suggestions by Del Brutto et al. (2017) (Table 1) CT based NCC diagnoses are divided into cystic lesions showing the scolex, lesions highly suggestive of, and those compatible with NCC. The term "definite NCC lesions" for cystic lesions showing the scolex, pathognomonic for NCC, will be used. Any cystic lesion without scolex, single or multiple ring or nodular enhancing lesions, and parenchymal brain calcifications are categorized as lesions highly suggestive of NCC. Active NCC is defined as any cystic lesions (with and without scolex) or lesions with ring enhancement. Parenchymal calcifications are classified as inactive (Nash et al., 2004; Nash et al., 2006). For the "reference diagnosis" of NCC, the research team will use a composite case definition according to the criteria suggested by Del Brutto et al. (2017) and Gabriël et al. (2012) (Table 2) that are based on clinical symptoms, neuroimaging, detection of antibodies and antigens in serum and epidemiological considerations, corresponding to absolute, major and minor criteria (Gabriël et al., 2012; Del Brutto et al., 2017).

Table 1. Diagnostic criteria for NCC according to Del Brutto et al. 2017

**Diagnostic criteria according to Del Brutto et al. 2017**
**Absolute criteria:**<br>• Histological demonstration of the parasite from biopsy of a brain or spinal cord lesion.<br>• Visualization of subretinal cysticercus.<br>• Conclusive demonstration of a scolex within a cystic lesion on neuroimaging studies.
**Neuroimaging criteria:**<br>**Major neuroimaging criteria:**<br>• Cystic lesions without a discernible scolex.<br>• Enhancing lesions.[a]<br>• Multilobulated cystic lesions in the subarachnoid space.<br>• Typical parenchymal brain calcifications.[a]<br>**Confirmative neuroimaging criteria:**<br>• Resolution of cystic lesions after cysticidal drug therapy.<br>• Spontaneous resolution of single small enhancing lesions.[b]<br>• Migration of ventricular cysts documented on sequential neuroimaging studies.[a]<br>**Minor neuroimaging criteria:**<br>• Obstructive hydrocephalus (symmetric or asymmetric) or abnormal enhancement of basal leptomeninges.
**Clinical/exposure criteria:**<br>**Major clinical/exposure:**<br>• Detection of specific anticysticercal antibodies or cysticercal antigens by well-standardized immunodiagnostic tests.[a]<br>• Cysticercosis outside the central nervous system.[a]<br>• Evidence of a household contact with T. solium infection.<br>**Minor clinical/exposure:**<br>• Clinical manifestations suggestive of neurocysticercosis.[a]<br>• Individuals coming from or living in an area where cysticercosis is endemic.[a]

a Operational definitions. Cystic lesions: rounded, well defined lesions with liquid contents of signal similar to that of CSF on CT or MRI; enhancing lesions: single or multiple, ring- or nodular-enhancing lesions of 10–20 mm in diameter, with or without surrounding edema, but not displacing midline structures; typical parenchymal brain calcifications: single or multiple, solid, and most usually < 10 mm in diameter; migration of ventricular cyst: demonstration of a different location of ventricular cystic lesions on sequential CTs or MRIs; well-standardized immunodiagnostic tests: so far, antibody detection by enzyme-linked immunoelectrotransfer blot assay using lentil lectin-purified T. solium antigens, and detection of cysticercal antigens by monoclonal antibody-based ELISA; cysticercosis outside the central nervous system: demonstration of cysticerci from biopsy of subcutaneous nodules, X-ray films or CT showing cigar-shape calcifications in soft tissues, or visualization of the parasite in the anterior chamber of the eye; suggestive clinical manifestations: mainly seizures (often starting in individuals aged 20–49 years; the diagnosis of seizures in this context is not excluded if patients are outside of the typical age range), but other manifestations include chronic headaches, focal neurologic deficits, intracranial hypertension and cognitive decline; cysticercosis-endemic area: a place where active transmission is documented.

b The use of corticosteroids makes this criterion invalid.

Table 2. Degrees of diagnostic certainty for NCC according to Del Brutto et al. 2017

**Degrees of diagnostic certainty according to Del Brutto et al. 2017**
**Definitive diagnosis:**<br>• One absolute criterion.<br>• Two major neuroimaging criteria plus any clinical/exposure criteria.<br>• One major and one confirmative neuroimaging criteria plus any clinical/exposure criteria.<br>• One major neuroimaging criteria plus two clinical/exposure criteria (including at least one major clinical/exposure criterion), together with the exclusion of other pathologies producing similar neuroimaging findings.<br>**Probable diagnosis:**<br>• One major neuroimaging criteria plus any two clinical/exposure criteria.<br>• One minor neuroimaging criteria plus at least one major clinical/exposure criteria.

3.3.2.3 Patient treatment and follow up (NCC and/or T)

Patients will also be informed about the results of their investigations during a follow up appointment at the health centre by the study doctor. Implications of possible NCC diagnosis will be discussed and, if indicated, treatment will be offered, according to national guidelines and Winkler's recommendations (Winkler, 2012, Winkler and Richter, 2015). Symptomatic NCC cases with active lesions will be treated with anthelminthic medication and steroids for 2-4 weeks according to the response. Upon initiation of treatment, patients will be admitted to the district hospital for a few days to ensure close supervision of potential side effects during treatment initiation and thereafter will be followed up at the rural health centre. At set time points a clinical examination and *T. solium* serological work up will be performed as described above. A second CT scan will be offered to monitor treatment success. Subsequent CT scans will be performed based on clinical decisions. Treatment of re-infection will be tailored to individual cases.

Epileptic seizures will be managed with available anti-epileptic medication and according to national guidelines (Winkler and Richter, 2015). Seizure semiology and CT scan result will influence the choice of the anti-epileptic medication and its withdrawal will be decided on an individual basis by the study neurologist. If the patient was already on anti-epileptic treatment

initiated elsewhere with satisfactory outcome, treatment will be maintained. Protocols for the management of incidental CT findings (e.g. mass lesions, cerebrovascular accident) will be developed by the neurology post-doc and the supervising neurologists together with doctors at the district hospital, and the medical staff of the health centre. Patients will be referred and medical care and follow-up will be assured according to routine procedures and current standard of care of the hospital. The latter will also be will be taught on information delivery and clinical follow-up of these patients in accordance with the local standard of care (WP2).

All efforts will be made to progressively refer all symptomatic patients (mainly people with epilepsy) to the local health facilities, where epilepsy care is free of charge. Previously asymptomatic people will be informed about any potential findings on their CT scans and will be briefed on potentially arising neurological symptoms/signs and the need of presentation to the nearest health facility.

Individuals with T will be treated with niclosamide (drug of choice, single dose - 2g orally) or praziquantel (single dose - 10mg/kg orally) according to the country's guidelines and availability of the drug. Niclosamide will be the drug of choice for T in people positive for T *and* (N)CC in order not to exacerbate symptoms of NCC. Household members of patients diagnosed with T, will have a medical follow up and treated if needed. They will be fully informed on potential symptoms/signs of NCC and the need of presenting to the nearest health facility should they become symptomatic.

3.3.3 Laboratory Procedures

3.3.3.1 POC test characteristics, production and quality assurance:

With this POC test, specific Abs against CC and/or T in serum or whole blood samples are captured by two well described recombinant antigens combined in one test: rT24H and rES33. The rES33 is based on an excretory-secretory protein of the adult tapeworm, and was previously evaluated in an immunoblot format, with a sensitivity of 99% and a specificity of 99.7% (Levine et al., 2007). The rT24H is a recombinant protein, which is based on a protein of the well-known specific 8-kD band area of the LLGP-EITB (present gold (reference) standard for CC-Ab detection) and was evaluated in an immunoblot format, with a sensitivity of up to 99% and specificity of 100% (Noh et al., 2014). In the present POC assay, rES33 and rT24H are coupled to 40nm gold particles using a passive adsorption gold conjugate protocol.

The rES33 and rT24H protein solutions are sprayed onto two nitrocellulose membranes (Sartorius CN140 unbacked, Göttingen, Germany) - which serve as the assay capture matrix - at 380 ng/mm and 200 ng/mm of protein respectively. Each of the two assay strips are further containing of a sample pad (Ahlstrom Cytosep 1662 (untreated), Helsinki, Finland), and a glass fibre conjugate pad (Ahlstrom 6613, Helsinki, Finland) which are held together using a backing card. The bound CC- and T-Abs are visualized by colloidal gold conjugate (OD 100) and Mouse IgG CGC (CGMIG-0600, Arista Biologicals Inc, Allentown PA, USA), OD50, which serve as antigen-Ab detecting cocktail imparting redish purple colour stripes on the membrane. In addition to the sprayed test antigens, Goat anti Mouse IgG (ABGAM-0500, Arista Biologicals Inc, Allentown PA, USA) is sprayed at 100 ng/mm as a control line, which serves as a quality assurance marker. Produced cards are cut into 3.5-mm individual strips by using a guillotine cutter and maintaining dry room humidity at no greater than 20% relative humidity. Strips are then placed into a double-strip standard cassette (Kinbio, Shanghai, Democratic Republic of China) (Figure 5).

Each POC test device is sealed separately in an aluminium pouch together with a desiccant. A kit contains ten devices sealed in a plastic bag together with a dropper bottle, chase buffer, micro-pipettors (2 pipettors/device), and a manual. Each kit will be labelled with a batch number, production date, and the following statement “For Evaluation Purposes Only”. Shipment of the POC kits will be performed at room temperature. Any storage of the kits exceeding three days should be performed at +4° C.

For the POC test evaluation in Zambia 2,400 POC tests will be produced in-house at CDC Atlanta in collaboration with Arista Biologicals Inc, Allentown PA, USA to meet high production standards. POC tests used in this project will be produced in three to four batches during the project period. Details concerning reagents and procedures of each batch will be documented according to laboratory standards which will be fully accessible to project monitors. For quality assurance, each batch will be pre-tested at CDC Atlanta with a defined positive and negative serum panel before shipment to Zambia (University of Zambia, School of Veterinary Medicine). From Zambia, kits will be distributed according to the work plan to the partner laboratories in Tanzania for field-evaluation at the study sites and in the RRL.

Field teams will be trained intensively in the handling and troubleshooting of the device. Full documentation on product specifications and transport will be made available. The compliance

on good storage and distribution practices along the supply chain will be documented. A detailed SOP will be written regarding POC test receipt, handling, storage, distribution, and retrieval of unused tests prior to implementation.

The developer´s team at CDC Atlanta will be available if POC test usage problems would arise.

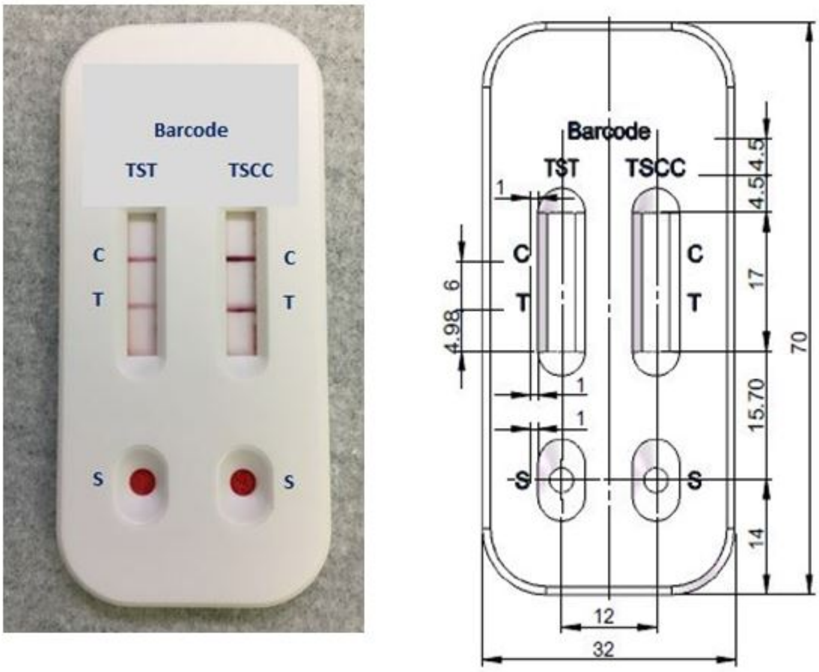

TST: *Taenia solium* taeniasis, TSCC: *Taenia solium* cysticercosis

C: Control line, T: Test line , S: Sample port

Figure 5: POC test device

3.3.3.2 Other laboratory procedures:

Field laboratory procedures, including the preparation of serum/ handling of stool samples, storage and shipment of samples have been detailed in a SOP.

Reference tests:

As there is no gold standard test for (N)CC or T diagnosis, multiple tests will be used to ensure a statistically sound estimation of the POC performance. All POC test positives and a subset of the POC test negatives (both CC and T) will be analysed with reference tests by the RRL for T/CC at the University of Zambia, a partner in this project. Additionally, a subset (20%) of all samples will be shipped to and analysed in the laboratories of ITM and SSI.

Also, a subset of the POC tests (10%) will be tested at the RRL (on serum).

Short description of the set of reference tests for CC:

- *LLGP-EITB*: For antibody detection the most specific and sensitive test and currently most widely used test is the enzyme-linked immunoelectrotransfer blot (LLGP EITB), an immunoblot of seven cysticercus glycoproteins, purified by lentil-lectin chromatography, with a Sp close to 100% and a sensitivity varying from 70% to 90%.
- *Ag-ELISA*: this test detects circulating Ag secreted/excreted by viable cysticerci in the serum. It has a Se of 90% and a Sp of 98% for the detection of active infections
- *rT24H– immunoblot test*: This immunoblot test makes use of one of the most promising recombinant Ag to detect CC-Ab with a demonstrated Se of up to 99% and a Sp of 100% (this is the same recombinant antigen as in the POC test)

Short description of the set of reference tests for T:

- *Copro-Ag ELISA:* This test detects adult worm Ag in stool samples. Its Sp and Se were estimated at 92% and 84%, respectively by Praet et al. (2013).
- *Copro-PCR*: this test is characterised by a Sp of 99% and a Se of 82%. The copro-PCR detects infections in a later stage than the copro-Ag ELISA.
- *16S/18S:* Ribosomal gene PCR products (amplified by general pro- and eukaryotic primers) are sequenced by ILLUMINA sequencing and data are analysed by the BIONmeta software (open source).
- *rES33 immunoblot test*: The rES33 immunoblot test is used for T-Ab detection in sera and shows a sensitivity of 99% and a specificity of 99.7% (Noh et al., 2014) (this is the same recombinant antigen as in the POC test)
- *Treatment and recovery*: all participants that test positive for T, will be treated (niclosamide or praziquantel), tapeworms will be recovered and confirmed by molecular tools (PCR-RFLP)

Description of diagnosis of NCC:

- CT scan based diagnosis of NCC will be divided into three groups, as described in CT "NCC diagnosis". The criteria proposed by Del Brutto et al. (2017) and Gabriël et al. (2012) will be used for diagnosis of clinical NCC. All participants/patients that tested

positive on any test will be treated as outlined above (4.2.2.3 Patient treatment and follow up).

3.4 Data Analysis

All test results and other data (demographic) from the community based study will be collected in a designated pre-prepared community based country specific study database (database A). Data from the references tests, clinical examinations and CT scans will be added to this database. Each participant/patient will be assigned a unique code. Good data management practices will be ensured.

All data analyses will be done by a qualified statistician, experienced in the evaluation of diagnostic tools. All data will be double entered independently in the databases, after checking data consistency (comparison and clean up), the data analysis will be performed using the commercial software packages, R (R Core Team (2016). R: A language and environment for statistical computing. R Foundation for Statistical Computing, Vienna, Austria. http://www.R-project.org/) and STATA (StataCorp. 2014. Stata Statistical Software: Release 13. College Station, TX: StataCorp LP).

Based on the study design, all selected samples from the communities will be tested with the POC test for both CC and T. All those testing positive in the POC test will be combined with 20% of those who test negative in the POC test to obtain the final sample that will be tested using the reference test. This final sample will be used to estimate the Se and Sp of the POC test for both CC and T using two approaches:

- A logistic regression (or Firth) modeling approach (Coughlin et al. 1992) that takes account of the results of the reference test and demographic information such as age, gender, occupation and region. This approach assumes that the reference test was used on the same sub-samples as the POC test.
- A conditional logistic regression approach that assumes that the results from the POC test and those from the reference test are not independent since they are from the same individual (paired sample). Similarly, demographic information such as age, gender, occupation and region will be added to the model.

In both approaches, Se and Sp will be derived from ROC curves of Se against 1-Sp.

For the evaluation the performance of the POC test for NCC, two sub-populations will be considered:

- ***POC Test positives with specific symptoms versus sub-sample (10%) of POC negatives:*** for this case, results of the CT scans will be crossed classified against those of the POC test and Se and Sp of the POC test will be calculated. The influence of demographic factors on the POC test performance for NCC will be assessed using a logistic regression (or Firth) modeling approach (Coughlin et al. 1992).
- ***POC Test positives with no specific symptoms versus sub-sample (10%) of POC negatives:*** In a similar manner, results of the CT scans will be crossed classified against those of the POC test and Se and Sp of the POC test will be calculated. In addition, the influence of demographic factors on the POC test performance for NCC will be assessed using a logistic regression (or Firth) modeling approach (Coughlin et al. 1992).

A mid-term analysis will be done after 350 samples for CC and at 700 samples for T have been collected, in order to re-estimate the sample size and make adjustments where necessary.

For CC and T, a Bayesian approach will be implemented to evaluate the performance of the POC test with respect to reference tests. The main output of all the afore-mentioned analyses will comprise of estimated Se and Sp of the POC test (adjusted for demographic factors) for the different scenarios and their 95% confidence limits. Positive and Negative predictive values will also be computed.

3.5 Data Management and Archiving

3.5.1 Data Management

Good data management practices will be ensured as sensitive data will be handled. Each participant/patient will be assigned a unique code.

Only one document with personal data linking the study participant name and location with the unique code will be made. This will be kept in lockable cupboards at study sites. A backup copy will be made and stored in a password protected database, only accessible by the study coordinator.

Biomedical samples will be labelled with a unique code for each participant, using indelible methods (permanent marker, or printed stickers) prior to or at the time of sample collection. All collection details and results of analyses will be checked and double-entered into a database, which will be regularly backed up to both cloud-based and external storage device systems, properly secured.

Questionnaire forms will be developed using an electronic mobile data collection software program (used also by MSF), and uploaded onto smartphones. Surveys will be translated into the local language and pre-tested with non-study participants prior to implementation. Questionnaire data will be captured directly onto the smartphones during the surveys, and downloaded into a database at the completion of each workday. The database will be regularly backed up to both cloud-based and external storage device systems. Questionnaire data will be back-translated into English by bilingual translators.

Rechargeable power packs and internet connection devices will be used in the field to ensure that the smartphones, laptops and databases necessary for the study are functional at all times.

3.5.2 Archiving

The Principal Investigator is responsible for ensuring a secure and appropriate location for storage of the Investigator's File and any other study related documentation present at site, as well as for ensuring that only site staff that is competent and delegated to work for the study has got access to the files.

Person identifier will be coded using unique ID number and will be entered into a central data base that will be password protected to secure maximum security.

CRFs with data on CT scans and treatment will also be kept in binders in locked up cup boards in a central office at the University of Zambia. Those data will in coded and uploaded to the central data base.

After study completion, all the relevant study documentation will be retained in accordance with the local legislation and should be retained for a minimum period of 10 years after completion of the study. The study will be performed according to standards of GCP, hence parts of the documents will also be archived at ITM. The ITM should be informed prior to destruction of the files.

The Investigator's File should at all times remain available for internal audits and/or inspections of regulatory authorities, also after completion of the project.

3.5.3 Open access to research data

Due to ethical and privacy concerns, individual participant data will not be made openly accessible after completion of the project. However, a data sharing managed access procedure will be adopted, by which researchers can request access to the data for specific secondary

analysis. Data may be shared for non-commercial research and/or provided that there are benefit sharing measures, with credit for the SOLID researchers.

3.6 Dissemination of results

Communication and publication of the study results regulations are described in the Grant Agreement and Consortium Agreement. Communication and publication of the study results will be carried out by the investigators jointly with the ITM. The ITM will be informed at least 3 months before disclosure of the data, in order to discuss the content of the presentation or manuscript. To safeguard privacy and confidentiality all published data will be anonymised. Due to ethical and privacy concerns, individual participant data will not be made openly accessible after completion of the project. However, a data sharing managed access procedure will be adopted.

The coordinator will organise an International Workshop at the end of the project to disseminate the final results. This workshop will include national stakeholders from Ministries of Health, Education, Fisheries and Livestock. Policy implications of the study findings will be presented and possible preventive strategies discussed.

At the preparatory stage the project will be presented and discussed with local communities and district and district hospital level personnel. Involvement of the educational and medical sectors will be secured.

Individual results will be communicated to the patients and if wished to share with the household. The overall findings from the study will be presented to the involved communities and suggestions for preventive measures discussed.

3.7 Ethical Issues

3.7.1 Ethical (and Regulatory) Review

This study will be submitted for formal review and approval to the Institutional Review Board of the ITM, Antwerp, Belgium, the Ethical Committee of the University Hospital in Antwerp, Belgium and the University of Zambia Biomedical Research Ethics Committee. No participants will be enrolled or participant related activities performed before written approval from these bodies is obtained.

Adherence to the principles of the most recent amended Declaration of Helsinki (Ethical Principles for Medical Research Involving Human Participants), ICH Good Clinical Practice Guidelines and applicable regulations and established international scientific standards will be ensured.

Approval will be sought from the National Health Research Authority, the Ministry of Health, Directors of Health in the district and the local community leaders. The project will be registered on the WHO/EDCTP Pan African Clinical Trial Registry.

Voluntary informed consent will be obtained from all human participants (or assent and parental or legally authorised representative consent for participants younger than 18 years of age) before enrolment in the study. The informed consent form will cover all aspects of the study, including participation in biomedical sampling, clinical examinations, CT scan and in the questionnaires. See further details in section 5.2 Obtaining Informed Consent.

Research activities will be conducted in a way which respects and protects, and is fair to, research participants and morally acceptable for the involved communities. The study will conform to accepted scientific principles, and as demonstrated in the contents of this proposal is based on adequate background knowledge of CC/T epidemiology and diagnostics. The study design adheres to strict epidemiological and diagnostic studies principles, which will ensure scientific validity. All investigators, sponsors and other participants are qualified individuals with requisite levels of education, expertise and experience to conduct their role competently. Qualified medical health professionals will perform all human sample collections and examinations.

Good data management practices will be ensured as sensitive data will be handled. Each participant/patient will be assigned a unique code.

Only one document with personal data linking the study participant name and location with the unique code will be made. This will be kept in lockable cupboards at study sites. A backup copy will be made and stored in a password protected database, only accessible by the study coordinator.

All biomedical samples will be assigned a study identification number (code) so that laboratory technicians are not able to identify individual participants. During data entry and analysis only coded data will be used. In case of SAE occurring the study medical doctor and monitor will access personal data (CRFs) to effect follow up. GCLP will reduce risk of potential harm to participants and laboratory staff. The study will be performed according to standards of GCP.

Hard and electronic copies of the data will be stored securely (locked cupboard in locked room). Only coded data will be shared with partner countries. Parts of the project documents will also be archived at ITM. Tanzania, Zambia and EU rules and regulations on data and sample export/import will be followed and all needed agreements (Data and material transfer agreements) will be obtained. Any data used in reports or publications will be presented anonymously and in such a way that prevents identification of individual participants by inference.

3.7.2 Obtaining Informed Consent

The informed consent procedure will describe the purpose of the study, the procedures (such as sampling, CT scanning and follow-up activities) to be followed, the risks and benefits associated with participation and obligations. The translated informed consent form (Appendix 7.4) will be pre-tested on non-study participants by bilingual members of the research team prior to the commencement of the study. For those individuals unable to read or write, the informed consent form will be read out aloud (in the local language).

Tactics such as repetition and explanation, and the answering of questions when they arise will be employed to ensure that every individual is thoroughly informed before choosing whether or not to participate in the study. Adequate time and resources will be made available for informed-consent procedures.

The study will be conducted in poor rural settings including participants living in low resources environment with access to limited health care and are considered a vulnerable group and great care will be exerted to prevent any coercion during the inclusion process.

Participants willing to participate in the study will be asked to provide written consent by signing the informed consent form. Inkpads will also be provided so that illiterate study participants can create a thumbprint on the form in substitution of a signature. For study participants younger than 18 years of age, verbal assent (or written if the minor is literate) and written consent from their parent or legally authorised representative will be obtained.

Study participants (or parents or legally authorised representatives) will be informed that participation in the study is completely voluntary and it will be made clear that the participant can withdraw from the study at any time without giving reasons, without any negative consequences and without affecting their accesses to treatment. They will also be informed that

the samples provided will be retained for longer than the end of the study and that they may be used for further research in the field.
Participants found positive for T will be offered treatment and participants with CC/NCC will be managed according to best practice.

Personal data (CRFs) will be kept in lockable cupboards at study sites. During data entry and analysis data will be coded. In case of SAE occurring the study medical doctor, the PI and monitor will access personal data (CRFs) to effect follow up. GCLP will reduce risk of potential harm to participants and laboratory staff. The study will be performed according to standards of GCP. Hard and electronic copies of the data will be stored securely (locked cupboard in locked room). Only coded data will be shared with partner countries. Parts of the project documents will also be archived at ITM. Tanzania, Zambia and EU rules and regulations on data and sample export/import will be followed and all needed agreements (Data and material transfer agreements) will be obtained.

3.7.3 Insurance

The Coordinator of this study, the Institute of Tropical Medicine has obtained a (no-fault) study insurance to cover any injury, damage or loss to study participants and the related study staff and which is caused directly or indirectly by their participation in the study.

3.7.4 Obligations of investigators

Investigators will:

- Refrain from unjustified deception, undue influence, or intimidation;
- Seek consent only after ascertaining that the prospective participant has adequate understanding of the relevant facts and of the consequences of participation and has had sufficient opportunity to consider whether to participate;
- Obtain a signed or thumb-printed form as evidence of informed consent from all participants;
- Renew the informed consent if there is significant changes in the conditions or procedures of the research;
- Ensure that all personnel working on the study comply with all aspects of the ethical considerations.

Compensation for participation

No payment or reimbursement shall be given for participation in the human blood sampling, faecal submission, clinical examinations. Diagnosis and treatment will be free of charge. Transport to and from the scanning facilities will be organised and will be free of charge. Incentives (e.g. light refreshments) may be provided.

3.7.5 Benefits, harms and risks of study participation

The direct benefits to participants in the study include receiving free diagnosis and treatment for T and (N)CC if required. Any harm to a participant as a result of participating in this study is not expected. Collection of blood samples by venepuncture poses minimal risk, and will be undertaken by qualified health professionals according to best practice procedures.

When undergoing a brain scan, the participants body will be subject to radiation, which when repeated often may harm specific organs. This however is not expected from a one- off examination. A trained radiology technician will inject contrast in the participant's vein during the examination. Contrast will not be given in case of thyroid or renal disease, this as well as the occurrence of allergic reactions under similar conditions will be asked to the participant prior to the investigation. However, in case of clinical suspicion (goiter, history of hypertension or diabetes, the study physician at the local hospital will perform a test. Even in healthy participants, there is still a chance that the body may react to the contrast injected. The participant may experience itching, chest tightness or hot flushes amongst other. If this is the case, the technician will immediately stop the investigation and will give specific medication. The chances of "allergic" symptoms are low. However, if they occur under similar circumstances we will only take a plain scan without injecting contrast. A specific training will be provided to the health professionals and clear management lines will be put in place in case of need. For these reasons children below the age of 10 years and pregnant women will be excluded. To rule out pregnancy, a systematic urinary pregnancy test will be systematically offered and performed to all women of reproductive age, and a specific SOP describing the patient flow will be implemented.

Safeguarding confidentiality

Data confidentiality shall be safeguarded by issuing participants with codes, which shall be linked to their samples and clinical examination CRFs, thereby blinding investigators as to the identity of the sample or information they are analysing. Identifiable data shall only be used for the allocation of treatments and shall only be submitted to investigators or databases if permission for that submission has been obtained from the identifiable individual.

Use of stored biological samples and related data

Voluntary informed consent will be obtained from all participants to store biological samples for future use within the field of *T. solium* diagnostics. The blood and faecal samples kept in ethanol/formalin will be stored at the University of Zambia and a subset of 20% of the samples will be sent to Denmark and Belgium for quality control with a material transfer agreement. The samples will be kept for 10 years at the recipient institutions and be destroyed once the 10 years have passed. The samples might be used for new diagnostic tests for *T. solium* infections as they become available, with an intended research rather than commercial goal. Any scientific research that may use the samples in the future will be approved by the National Health Research Authority. The study will be performed according to standards of GCP. Hard and electronic copies of the data will be stored securely (locked cupboard in locked room) at the School of Veterinary Medicine at the University of Zambia. Only coded data will be shared with partner countries.

Parts of the project documents will also be archived at ITM.

Disclosure and review of potential conflicts of interest

There are no perceived conflicts of interests for the research.

Deviations

Due to unforeseen circumstances, it may be necessary to substantially deviate from the study protocol. Should this be the case a substantial amendment to the study protocol will be formally submitted for approval to the IRB and ethics committees before the implementation of the changes made in the protocol.

A mid-term evaluation will be conducted when predetermined sample has been obtained (see 4.1.1 Sample Size and Power). Results will be discussed with the General Assembly and the

External Expert Advisory Board (EEAB) and the based on the results an amendment to the protocol can be proposed.

3.8. Monitoring and Quality Control

The coordinator supported by the management support team (MST) consisting of all (co)WP-leaders will put in place a monitoring and evaluation plan (approved by the general assembly (GA)) to ensure this study achieves its stated objectives within the specified timeframes and the work carried out is of the highest possible quality and ethical standard. Completion of activities and progress towards critical milestones for each objective will be monitored to ensure these are completed in the appropriate timeframes. This will be done by having a well-defined supervisory structure (Figure 6). Responsibility for each objective activity will be allocated to a specified WP team member and progress of each activity against the timelines will be reviewed on a monthly basis.

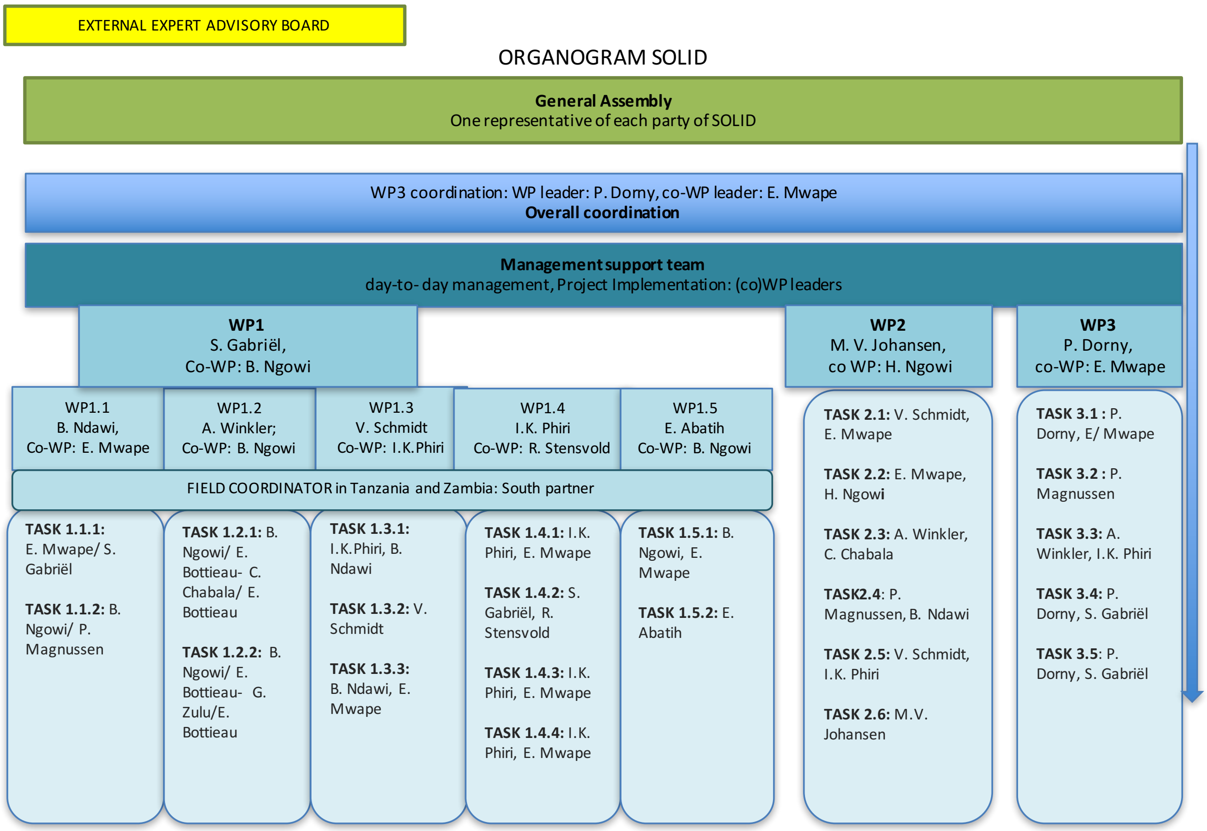

Figure 6: SOLID organogram, referring to the tasks presented in Figure 1

The coordinator will ensure that the study protocol is accepted by the relevant IRBs and ECs before the start of the field studies, that informed consent will be obtained from all study participants/patients, and that the interest and well-being of all study participants/patients is safeguarded.

All study staff will be duly qualified by training or experience for the tasks they perform in the study. Additionally, before the initiation of the project the staff will be trained on the protocol, the IC forms, the use of the POC test and GCLP, on CRFs, double data entry and GCP and on patient management and treatment (WP2).

An EEAB will be established, consisting of two experts on *T. solium* diagnostics that will evaluate the project progress and give advice on the mid-term evaluation.

Any unforeseen constraints to completion of activities will be identified and remedial actions taken in a timely manner. A fast track system for major protocol deviations/violations will be put in place. Progress of all objectives will be reviewed at a program review (held via Skype or teleconference) three times/year and during the yearly physical meetings. Any outstanding issues can be reviewed and solutions generated by the whole team with possible consultation of the EEAB. In specific high activity periods concerning one partner, more regular exchanges will take place between the specific partners. As such, the execution of the project will be monitored internally by the management team.

The coordinator will maintain contact with the POC test providers, CDC. The coordinator will also be responsible for the drawing up of formal contracts/agreements between project partners, and ensure that appropriate internal and external quality controls are set up (audits).

The local centre will allow the ITM's monitor or a monitor designated by the ITM access to any study-related files or source document present at the local centre.

A mid-term performance analysis of the POC test will be performed halfway through the studies (when a sufficient amount of data is available, see above) and will be communicated to the EEAB. Based on the results of this analysis the protocol will be maintained or adapted. In case a substantial amendment to the study protocol is needed this will be formally submitted for approval to the IRB and ethics committees before the implementation of the changes made in the protocol.

Safety

An external expert covering the role of the safety monitor will be identified. The monitor will have clearly designated tasks related to the potential SAE related to the blood sampling procedure, treatment and CT scan performance. The study doctor on site will examine, record and manage any SAE including a close active follow up, according to pre-established procedures. The study doctor will report SAE to the PI (Dr G. Zulu) and the monitor; the monitor will subsequently inform the relevant authorities (including the IRB and EC's, sponsor and manufacturer) within a predefined period of time. Appropriate, free medical care, transport and stay at the hospital if necessary and follow-up will be assured in case of study related SAE. However, these are not expected as patient management and treatment following diagnosis will be performed according to routine procedures of the DH. Blood samples by venipuncture poses minimal risk, and will be undertaken by qualified health professionals. Allergic reactions from a one-off CT scan examination are very rare and their specific urgent management will be consolidated and reinforced by the research team for the (experienced) radiological staff and the supervising radiologist."

4.0 BUDGET

The whole consortium (Zambia, Tanzania, Belgium, Germany and Denmark) budget is of 1,889,999 euro.
The study budget attributed to the Zambian component of patient recruitment is detailed below:

SOLID Project Budget Summary in Euro

	**Category**	**Period 1**	**Period 2**	**Period 3**	**Period 4**	**Total**
A	Personnel - unit cost	27,722.00	72,507.00	43,977.00	30,138.00	154,244.00
B	Travel	2,463.00	6,854.00	3,301.50	2,548.00	15,166.50
C	Equipment	63,235.89	25,282.94	12,522.22	2,582.25	59,960.43
D	Sampling, laboratory consumables	14,357.00	71,848.00	67,540.00	2,686.00	154,431.00
E	Contingency	1,000.00	2,000.00	2,000.00	1,200.00	6,200.00
F	Total Direct costs	108,777.89	178,491.94	129,340.72	39,154.25	383,801.93
G	Indirect costs (5% of direct costs)	5,725.15	9,394.31	6,807.41	2,060.75	95,950.48
H	**Total Estimated Cost**	**114,503.04**	**187,886.25**	**136,148.13**	**41,215.00**	**479,752.41**

5.0 PROJECT TIMELINES

			YEAR 1 Sept '16 – Aug '17				YEAR 2 Sept '17 – Aug '18				YEAR 3 Sept '18 – Aug '19				YEAR 4 Sept '19 – Aug '20			
**Specific objectives**	**WP**	**Tasks**	**Q1**	**Q2**	**Q3**	**Q4**	**Q1**	**Q2**	**Q3**	**Q4**	**Q1**	**Q2**	**Q3**	**Q4**	**Q1**	**Q2**	**Q3**	**Q4**
To assess the test performance to diagnose T and (N)CC at community level	**WP1 POC TEST EVALUATION**	See description																
To conduct community based field studies	**WP1.1 Field studies**	Task 1.1.1 Conduct community based field studies																
To conduct clinical and CT work-up of selected patients	**WP1.2 CT Scan – Patient management**	Task 1.2.1 Clinical work-up and CT scan examination																
To establish NCC diagnosis and ensure appropriate treatment																		
To ensure adequate patient follow-up and referral to the hospital		Task 1.2.2 Patient treatment and follow-up																
POC test production, monitoring and delivery to the study partners	**WP1.3 CDC test delivery**	Task 1.3.1 Pilot testing of POC test in Zambia RRL and development of standard operating procedures																
		Task 1.3.2 Monitoring of POC test production at CDC and distribution to study partners																
Ongoing monitoring and quality management		Task 1.3.3 Ongoing monitoring and quality control of POC performance																
To analyze all POC test positive and a subset of the POC negative samples with specific tests for (N)CC/T	**WP1.4 Reference diagnostic tests**	Task 1.4.1 Analyses of samples in RRL																
		Task 1.4.2 Analyses of subset of samples in Belgium and Denmark																
To analyze a subset of the samples with POC test at the RRL and selected laboratories in Belgium and Denmark		Task 1.4.3 Compilation of all RRL information supporting NCC diagnosis																
		Task 1.4.4 Transfer of the laboratory test results to WP1.2 and WP1.5																
To prepare all needed databases and follow up data entry	**WP1.5 Data management, Analyses**	Task 1.5.1Data management setup and follow up																

To statistically analyse the community based data		Task 1.5.1 Data analyses																
To transfer POC to the study sites and train health professional in using POC in the field studies	**WP2 TRAINING AND TECHNOLOGY TRANSFER**																	
To improve the capacity to understand T/(N)CC risk and control		Task 2.2 Train health professionals in disease risk and control																
To improve the capacity to manage T/(N)CC patients at rural health centre		Task 2.3 Train health professionals in management of T/NCC patients																
To strengthen capacity to conduct diagnostic and clinical studies at the partner institutions		Task 2.4 Improve capacity to conduct diagnostic and clinical studies at the partner institutions in the South																
To improve diagnostic capacity and GCLP of the regional reference laboratory		Task 2.5 Improve diagnostic capacity and GCLP of the regional reference laboratory for T/CC																
To supervise post graduate students		Task 2.6 Supervise PhD and Master Students																
To coordinate/monitor all WP's and activities (including quality control)	**WP3 CO-ORDINATION**	Task 3.1. Coordination																
To organise regular meetings (via skype) and yearly physical team meetings		Task 3.2. Monitoring and quality control																
To maintain the communication with the POC test developers (CDC)																		
To provide a detailed WHO endorsement and implementation plan if the tool is successful, and to facilitate commercialisation		Task 3.4 Endorsement and implementation																
To link with Cysticercosis Working Groups and other stakeholders (Networking)		Task 3.3 Networking																
To organise an International Workshop for the dissemination of the final results		Task 3.5 International Workshop																

6.0 REFERENCES

1. Banoo S, Bell D, Bossuyt P, Herring A, Mabey D, Poole F, Smith PG, Sriram N, Wongsrichanalai C, Linke R, O'Brien R, Perkins M, Cunningham J, Matsoso P, Nathanson CM, Olliaro P, Peeling RW, Ramsay A, TDR Diagnostics Evaluation Expert Panel. Evaluation of diagnostic tests for infectious diseases: general principles. Nat Rev Microbiol. 2010: 8(12 Suppl): S16-28. doi:10.1038/nrmicro1523
2. Biesheuvel CJ, Vergouwe Y, Oudega R, Hoes AW, Grobbee DE, Moons KG. Advantages of the nested case-control design in diagnostic research. BMC Med Res Methodol. 2008: 8: 48. doi: 10.1186/1471-2288-8-48
3. Blocher J, Schmutzhard E, Wilkins PP, Gupton PN, Schaffert M, Auer H, Gotwald T, WHO, Assembling a framework for intensified control of taeniasis and neurocysticercosis caused by *Taenia solium*; report of an informal consultation. 2014. http://apps.who.int/iris/bitstream/10665/153237/1/9789241508452_eng.pdf?ua=1
4. Braae, UC, Saarnak, CFL, Mukaratirwa, S, Devleesschauwer, B, Magnussen, P, Johansen, MV. *Taenia solium* taeniosis/cysticercosis and co-endemicity with schistosomiasis in Africa. Parasite Vector. 2015: 8: 323. doi: 10.1186/s13071-015-0938-7.
5. Branscum AJ, Gardner IA, Johnson WO. Estimation of diagnostic-test sensitivity and specificity through Bayesian modeling. Prev Vet Med. 2005: 68 (2-4): 145-163. doi:10.1016/j.prevetmed.2004.12.005
6. Bristwo et al. 2014. Multisite Laboratory Evaluation of a Dual Human Immunodeficiency Virus (HIV)/Syphilis Point-of-Care Rapid Test for Simultaneous Detection of HIV and Syphilis Infection. Open Forum Infect Dis
7. Corstjens PL, de Dood CJ, Priest JW, Tanke HJ, Handali S, Cysticercosis Working Group in Peru. Feasibility of a lateral flow test for neurocysticercosis using novel up-converting nanomaterials and a lightweight strip analyzer. PLoS Negl Trop Dis. 2014: 8(7):e2944. doi: 10.1371/journal.pntd.0002944

8. Coughlin SS, Trock B, Criqui MH, Pickle LW, Browner D, Tefft MC. The logistic modeling of sensitivity, specificity, and predictive value of a diagnostic test. J Clin Epidemiol. 1992 Jan;45(1):1-7.

9. Del Brutto OH. 2012. Diagnostic criteria for neurocysticercosis, revisited. Pathog Glob Health. 106:299-304.

10. Del Brutto, O.H., Nash, T.E., White, A.C., Jr., Rajshekhar, V., Wilkins, P.P., Singh, G., Vasquez, C.M., Salgado, P., Gilman, R.H., Garcia, H.H., 2017. Revised diagnostic criteria for neurocysticercosis. J. of the neurological sciences 372, 202-210.

11. FAO/WHO [Food and Agriculture Organization of the United Nations/World Health Organization]. 2014. Multicriteria-based ranking for risk management of food-borne parasites. Microbiological Risk Assessment Series No. 23. Rome. 302pp

12. Gabriël, S., Blocher, J., Dorny, P., Abatih, E.N., Schmutzhard, E., Ombay, M., Mathias, B., Winkler, A.S., 2012. Added Value of Antigen ELISA in the Diagnosis of Neurocysticercosis in Resource Poor Settings. PLoS Negl. Trop. Dis. 6, e1851.

13. Handali S, Klarman M, Gaspard AN, Dong XF, Laborde R, Noh J, Lee YM, Rodriguez S, Gonzalez AE, Garcia HH, Gilman RH, Tsang VC, Wilkins PP. Development and evaluation of a magnetic immunochromatographic test to detect Taenia solium, which causes taeniasis and neurocysticercosis in humans**.** Clin Vaccine Immunol. 2010: 17: 631-637.

14. Levine MZ, Calderón JS, Wilkins PP, Lane WS, Asara JM, Hancock K, Gonzalez AE, Garcia HH, Gilman RH, Tsang VCW. Characterization, cloning, and expression of two diagnostic antigens for *Taenia solium* tapeworm infection. J Parasitol. 2004: 90 (3): 631–638. doi: http://dx.doi.org/10.1645/GE-189R

15. Levine MZ, Lewis MM, Rodriquez S, Jimenez JA, Khan A, Lin S, Garcia HH, Gonzales AE, Gilman RH, Tsang VC. Development of an enzyme-linked immunoelectrotransfer blot (EITB) assay using two baculovirus expressed recombinant antigens for diagnosis of *Taenia solium* taeniasis. J Parasitol. 2007: 93, 409–417.

16. Mwape KE, Phiri IK, Praet N, Muma JB, Zulu G, Van den Bossche P, de Deken R, Speybroeck N, Dorny P, Gabriël S. *Taenia solium* infections in a rural area of eastern Zambia—a community based study. PLoS Negl Trop Dis. 2012: 6 (3): e1594. doi: 10.1371/journal.pntd.0001594

17. Mwape KE, Phiri IK, Praet N, Speybroeck N, Muma JB, Dorny P, Gabriël S. The incidence of human cysticercosis in a rural community of eastern Zambia. PLoS Negl Trop Dis. 2013: 7 (3): e2142. doi: 10.1371/journal.pntd.0002142

18. Mwape KE, Blocher J, Wiefek J, Schmidt K, Dorny P, Praet N, et al.(2015). Prevalence of Neurocysticercosis in People with Epilepsy in the Eastern Province of Zambia. PLoS Negl Trop Dis 9 (8): e0003972.doi:10.1371/journal.pntd.0003972

19. Ndimubanzi PC, Carabin H, Budke CM, Nguyen H, Qian YJ, Rainwater E, Dickey M, Reynolds S, Stoner JA. A systematic review of the frequency of neurocysticercosis with a focus on people with epilepsy. PLoS Negl Trop Dis. 2010: 4 (11): e870. doi: 10.1371/journal.pntd.0000870

20. Noh J, Rodriguez S, Lee YM, Handali S, Gonzalez AE, Gilman RH, Tsang VC, Garcia HH, Wilkins PP. Recombinant protein- and synthetic peptide-based immunoblot test for diagnosis of neurocysticercosis. J Clin Microbiol. 2014: 52 (5): 1429–1434. doi: 10.1128/JCM.03260-13

21. Praet N, Rodriguez-Hidalgo R, Speybroeck N, Ahounou S, Benitez-Ortiz W, Berkvens D, Hul AV, Barrionuevo-Samaniego M, Saegerman C, Dorny P. Infection with versus exposure to *Taenia solium*: what do serological test results tell us? Am J Trop Med Hyg. 2010: 83(2): 413-415. doi: 10.4269/ajtmh.2010.10-0121

22. Rutjes AW, Reitsma JB, Vandenbroucke JP, Glas AS, Bossuyt PM. Case-control and two-gate designs in diagnostic accuracy studies. Clin Chem. 2005: 51 (8):1335-1341. doi: 10.1373/clinchem.2005.048595

23. STARD, 2015. http://www.equator-network.org/reporting-guidelines/stard/

24. Tsang VC, Brand JA, Boyer AE. An enzyme-linked immunoelectrotransfer blot assay and glycoprotein antigens for diagnosing human cysticercosis (*Taenia solium*). J Infect Dis. 1989: 159 (1): 50–59. doi: 10.1093/infdis/159.1.50

25. WHO, 2013. Sixty-sixth World Health Assembly (WHA66.12). Tech. rep., World Health Organisation, Geneva.

26. WHO, Assembling a framework for intensified control of taeniasis and neurocysticercosis caused by *Taenia solium*; report of an informal consultation. 2014. http://apps.who.int/iris/bitstream/10665/153237/1/9789241508452_

27. Winkler AS, Blocher J, Auer H, Gotwald T, Matuja W, Schmutzhard E. Epilepsy and neurocysticercosis in rural Tanzania – an imaging study. Epilepsia. 2009: 50 (5): 987-993. doi: 10.1111/j.1528-1167.2008.01867.x

28. Winkler AS. Neurocysticercosis in sub-Saharan Africa: a review of prevalence, clinical characteristics, diagnosis, and management. Pathog Glob Health. 2012: 106: 261-274. doi: 10.1179/2047773212Y.0000000047

29. Winkler AS, Richter H. Landscape analysis: management of neurocysticercosis with an emphasis on low- and middle-income countries. 2015 2015. Report No.: Contract No.: WHO/HTM/NTD/NZD/2015.05.

7.0 APPENDICES

7.1 Information sheet in English

RESEARCH PARTICIPANT INFORMATION SHEET

SOLID: Evaluation of a simple test for the diagnosis of pork tapeworm infection

Investigators:	Dr. G. Zulu and Dr. K.E. Mwape
Organizations:	Ministry of Health, Zambia and University of Zambia
Sponsor:	Institute of Tropical Medicine, Antwerp, Belgium
Funders:	EDCTP (European and Developing Countries Clinical Trials Partnership) and BMBF (German Federal Ministry of Education and Research)

You are/your child is invited to take part in a research study on the disease caused by the pork tapeworm which is common is this area. Before you decide to be part of this study, it is important that you understand the information in this form, because it explains your rights and our responsibilities to you. In this information and consent form, the purpose, examinations, possible advantages, risks and inconveniences related to this study and your right to stop your participation at any time are explained. You have the right to ask questions at any time to the doctors in charge of this study. Your participation is completely voluntary. You may talk to anyone you feel comfortable with about the research and you can take your time to think about whether you want to participate or not. With you, we mean either you or your child throughout the document.

PURPOSE AND DESCRIPTION OF THE STUDY

This research project is undertaken to learn more about a new test to diagnose the disease caused by the adult pork tapeworm that can live in your intestines and its immature worm that can live in your brain and muscles. The adult pork tapeworm can give stomach pain and diarrhoea and the immature worm can cause epilepsy and severe headache.

We want to see how good the new and simple test is at finding people with the adult pork tapeworm and also those with the immature worm. Around 1200 participants will take part in this study.

HOW THE STUDY IS DONE

If you join the study, and if you meet the conditions to participate, you will be first asked to give a drop of blood using a finger prick to be placed on the new simple test. If the simple test is positive for either the adult pork tapeworm or immature worm you will be asked for a blood sample that will be taken using a needle prick and asked to give a stool sample using an appropriate container. Even when you are negative, you may also be asked to provide a blood and stool sample using the appropriate container. This is important to see how well the new simple test works and confirm our findings. If you are positive for the immature worm, you will receive further examination and be taken to a hospital either in Chipata or Lusaka for a scanning of the brain. Some individuals with negative results will be chosen at random and also asked to undergo a scanning of the brain as the immature worm in the brain often do not cause symptoms/signs and may even show a negative test result. Details on the brain scanning are given below.

In case you are infected with the pork tapeworm, you will be asked to visit the closest rural health centre to get treatment by the study staff at the health centre.

The blood and stool samples will be will be submitted for additional tests in Denmark and Belgium. From the questions, we want to find out what you know about this disease and the condition of your household that might favour spread of the disease.

We will write your name, but will only use it if you need treatment or follow-up. If you are infected with the adult or immature pork tapeworm, you will receive treatment and medical follow-up at the rural health centre or hospital free of charge.
You will be asked to be part of this study for the whole duration of the study.

Storage of samples
We would like to store a part of the blood/stool samples taken from you during the study at the University of Zambia for 10 years. This blood/stool samples might in the future be used for other tests related to the tapeworm disease. Any scientific research that may use your samples in the future has to be approved by the National Health Research Authority. In any case, your personal information like your name will never be shared with anyone outside of the study team or be published anywhere. Also, your samples will never be sold or used for commercial purposes.

If you change your mind, you can withdraw this consent for sample storage at any time (for you or your child). You may ask Dr. K. E. Mwape at any time (tel. numbers below).

You can indicate whether or not you agree with this future research on the last page of this document. Even if you decide that you do not want us to store your blood and stool sample, you can still participate in the study.

RISKS AND INCONVENIENCES
Taking a blood sample may cause some discomfort, bleeding or bruising where the needle enters the body (1% risk) or there may be swelling in the area. Very rarely, fainting, local infection happens or the bleeding will not stop. Blood sampling will be conducted by qualified health professionals, and care will be taken to prevent any problems and reduce the discomfort as much as possible. If you or your child experience anything unusual around the needle prick please do not hesitate to visit the rural health centre straight away for a check-up.
When undergoing a brain scan, your body will be subject to radiation, which when repeated may cause harm to specific organs of your body. This however is not expected from just one examination. The radiology technician will place an intravenous line (a thin, flexible, plastic hose) in your forearm and inject a fluid in your vein during the scan which will help the chance to see whether there is anything wrong with your brain. You should not get this fluid, if you are aware of having a thyroid or kidney disease, which you have to let us know before the radiology. There is a very small risk that you develop an allergic reaction to the fluid. You may experience itching, chest tightness or hot flushes. A strange feeling of body heating is quite common but not dangerous. In any case, you have to let the technician know immediately, and he will assess if the test should continue or not, and if you require some immediate treatment for the reaction. Should you have experienced such a reaction in a previous scanning, you should let us know, as there is a risk that this may happen again. In that case, we would take a scan without injecting the fluid.

BENEFITS
You will benefit from the study, as you will receive diagnosis and a treatment against tapeworm infections free of charge. In addition, through the scanning of your brain you will get important information about having any immature worm in the brain. The study is also expected to benefit the community as it will reduce the overall occurrence of the diseases in the community, and will hopefully reduce the chance of other people getting infected or you getting infected again. You will not receive payment for participating in the study but transport to and from the hospital either in Chipata or Lusaka will be taken care of.

COMPENSATION AND INSURANCE
All examinations related to you taking part in the study are free of charge to you. The organizer of this study, the Institute of Tropical Medicine, has obtained an insurance to cover any possible harm or injury that may be caused by participation in the study. If you get harmed or have questions about injuries as a result of being in the study, please contact the responsible researcher or doctor in the health centre.

PROTECTION OF YOUR PRIVATE LIFE
We will do everything we can to protect your privacy. Any information about you will be stored in an electronic database, and we will only use a code and not your name. The documents where your name is mentioned will not be shared with anyone, except the study researchers, study doctor and few other people who have to keep it confidential, such as representatives of the Institute of Tropical Medicine. By signing this informed consent form you agree with this access to your records.
The findings of the study will be published in medical journals and (coded) data can be shared in an international database. Your name will not appear in any database, report or publication resulting from this study.

ETHICS COMMITTEE
Before the start, this study was reviewed and approved by the University of Zambia Biomedical Research Ethics Committee, the Ethical Committee of the University Hospital in Antwerp, Belgium and by the Institutional Review Board of the Institute of Tropical Medicine, Antwerp, Belgium. These Ethics Committees also do on-going review of the study to make sure it is carried out in the safest way possible.

VOLUNTARY PARTICIPATION
Your participation in this study is entirely voluntary. It is your choice whether you want to take part in it or not.
You also have the right to stop your participation in the study at any time, even after you have signed this Informed Consent Form. You do not have to give a reason for wanting to stop being part of the study. This will not affect you or other household members or the community in any way.
The study researcher can stop your participation in this study at any moment as well, even without your permission, if he/she judges this in your best interest or if you do not follow the instructions for participation in the study despite several reminders.

CONTACT PERSON IN CASE OF QUESTIONS
If you have any questions concerning your participation in this study, your rights or if you think you have been harmed as a result of the study, you can contact, now, during, or after the study:

Dr. Gideon Zulu, Ministry of Health, Government of the Republic of Zambia. Cell: +260 976116556. E-mail: gideonzulu@yahoo.com

Kabemba Evans Mwape, Department of Clinical Studies, School of Veterinary Medicine, Box 32379, Lusaka. Tel: +260 211291515, Cell: +260 977819236. E-mail: kemwape@yahoo.com or evans.mwape@unza.zm

The Chair person, University of Zambia, Biomedical Research Ethics Committee, Ridgeway campus, P.O. Box 5110, Lusaka. Tel: +260 211 256067 or E-mail: unzarec@unza.zm

7.2 Information sheet in Chewa

PEPALA YA UTHENGA KWA OTENGA MBALI MU KUFUFUZA

SOLID: Kufufuza kwa kapimidwe kofewa mukuzindikira matenda ya minyoka zo chokera ku nkhumba

Ofufuza: Dr. G. Zulu and Dr. K.E. Mwape
Zigawo zomwe akuimilirako: Ministry of Health, Zambia and University of Zambia
Bungwe yo watandizira: Institute of Tropical Medicine, Antwerp, Belgium
Bungwe yo wapasa ndalama: EDCTP (European and Developing Countries Clinical Trials Partnership) and BMBF (German Federal Ministry of Education and Research)

Imwe/kapena mwana wanu mwaitanidwa kutengako mbali mu kufufuza kwa kapimidwe kofewa ka matenda obweletsedwa ndi minyoka ya mkhumba yomwe ikumapezeka kwambiri ku mzinda wakuno. Musanavomere kutengako mbali mukufufuza uku, ndichofunikira kuti muzindikire zolembedwa mu pepala iyi chifukwa ikufotokoza ma ufulu omwe muli nao ndiponso ma udindo athu kwa imwe. Mu pepala ya uthenga iyi, cholinga cha kufuza uku, kucencenta, ma phindu kapena zopezamo, ziopsezo ndi zovuta zomwe mungakumane nazo Kamba kakufufuza uku, ndiponso ma ufulu anu akuleka kutengako mbali mukufufuza uku pa ntawi ili yonse afotokozedwa. Muli ndi ufulu ofunsa mafunso kuli ba sing'anga oyanganira kufufuza uku. Kutengako mbali kwanu ndikozifunira, ndi kosakakamizidwa Mungalankule ndi munthu aliyense omwe mukumva ufulu olankula naye ndiponso mungalingalirepo pa nkaniyi musanasanke kutengamo mbali kapena kusatengamo mbali.

Mau akuti "Inu" mu pepala iyi, akutanthauza inu kapena mwana wanu.

CIFUKWA NDI KUFOTOKOZA KWA KUFUFUZA UKU

Kufufuza uku kukucitidwa ndi cholinga chakuyesa kapimidwe katsopano kofewa kopimira matenda obweretsedwa ndi minyoka za mkhumba zomwe zimakhala mumimba ya anthu ndiponso tiana take timakala mu bongo ndi nyama ya antu. Anthu ali ndi minyoka za m'mimba atha kumva mumimba kuwawa ndiponso kuthurula, ndiyeno ali ndi tiana taminyoka angankale aku khunyuka ndiponso kumva muthu kuwawa.
Tikufuna kuona ngati kapimidwe katsopano kofewa aka ndi kabwino mukudziwa anthu ali ndi minyoka kapena tiana take. Tikufuna anthu ofika 1200 kutengamo mbali.

MWAMENE KUFUFZA UKU KUZACITIDWA

Ngati imwe/mwana wanu (ndi chivomezi chanu) muvomera kutengako mbali mukufufuza uku, ndipo muvomelezedwa, tizaphempha kutengako magazi ochepekera pa chala ndikuwaika pa kopimira magazi. Ngati kopimira ka tsopano aka kapeza kuti muli ndi minyoka kapena tiana take, tizaphempha kutengako magazi ndi chimbudzi (tuvi) Nthawi zina, ngankale kuti simunapezeke ndi minyoka kapena tiana take, muzapempedwa kupatsa magazi ndi chimbudzi (tuvi). Ici ndi cofunikira po ona bwino mumene kapimidwe katsopano kofewa aka kakusebenzera. Mukapezeka ndi tiana ta minyoka, asing'anga azapitiriza kufufuza mwakupima thupi lanu ndiponso muzapelekedwa ku chipatala cacikulu ku Chipata kapena ku Lusaka komwe ubongo wanu uzajambulidwa pa makina ochedwa CT Scan. Ena otengamo mbali mukufufuza uku omwe sazapezeka na matenda alionse pambuyo popimidwa ndi kupima kwatsopano kofewa

azasankidwa kupita kukupimidwa kwa kujambula cithunzi ca ubongo chifukwa chakuti tiana ta minyoka situmaonetsa matenda mu munthu kwa nthawi zonse.

Kufotokoza kotheratu pa pakujambura chitunzhi ca ubongo kwapatsidwa pansipa.

Mukapezeka ndi minyoka za m'mimba, muzapempendwa kupita ku cipatala komwe muzaciritsidwa mwaulele. Magazi ndi chimbudzi(tuvi) azapitisidwa kukupimidwa kopambana ku maiko akunja ku Denmark ndi Belgium. Pomwe tizayamba kufunsa mafunso kwa banja lanu, tikufunabe kudziwa zomwe muzindikira pa matenda awa ndiponso mikalidwe ya pa nyumba panu yomwe ingathekese kupatsana kwa matenda awa pakati panu. Tizankhala kulemba maina anu omwe anga gwiritsidwe nchito mutafunika kuciritsidwa kapena kusatiridwa. Ngati mwapezeka na minyoka za m'mimba kapena tiana take, muzaciritsidwa mwaulele ndipo akaswili apa cipatala cacin'gono ku mzinda wanu ngankale aku cipatala cacikulu, azakusamalirani mwaulele kufikila kutha kwa m'dandanda wa makwala omwe muzapatsidwa.
Muzapempedwa kukhala mbali ya kufufuza kufikira ku mapeto

KASUNGIDWE KA MAGAZI NDI CHIMBUDZI/CHIMBUDZI/TUVI

Tikufuna kusunga gawo la magazi ndi chimbudzi zochokera kwainu ku the University Of Zambia padzaka khumi. Mutsogolo zingazagwiritsidwe nchito mumapimilo okudza matenda a miyoka za m'khumba. Magazi/chimbudzi anu angasebenzedwe kuntawi yakusogolo kuyanka zinanguso zofufuza. Kufufuza kulikonse kumene kuzafunika kugwiritsa nchito zomwezi kuzapelekedwa kuti adzione ndi kuzilola ku National Health Research Authority. Zonse zokhuza kuti imwe mudziwidwe zidzasungidwa mwachinsinsi ndi motetetzedwa ndipo kulibe zilizonse zaizo zimene zizagawidwa ndi munthu wina aliyense asali mukufufuza uku kapenanso ufalisidwa kuli konse. Magazi ndi chimbudzi yanu sizizagulitsidwa kapena ayi. Ngati mwasintha nzeru, mukhoza kuchokamo kapena kulesa chilolezo/kuvomela kwanu kuti zinthu zanu zisungidwe pakutumila a Dr. E. Mwape pa phone number 097 819236/096 752686. Ngakhale mwachita izo, mwakana kuti zinthu zanu zisungidwe mungakhale mukalimo ndithu mukufufuza kwakulu.

*Panthawi imene otenga mbali afuna kuziwa chilichonse kuchokera kuofufuza kapena ku Research Ethics Committee, ma adelesi awo mukhoza kuwapeza *pansi pa papelayi.*

ZIOPSEZO NDI ZINTHU ZOVUTHA

Kutenga magazi kungabwelese unsamvela bwino pang'ono chabe chifukwa chakuti zinganu/nyeleti ingakhetse timagazi kapena kummyuka pamene ilowa muthupi. Timagazi touma tungapangike pamene zinganu idalowa kapena pangakhale utupa pang'ono. Paichi ndi chapatali kuti munthu afoka, kugwa kapena kumayambitsa matenda. Kuchosa magazi kuzachitidwa ndi asing'anga kapena ogwila nchito muchipatala ndipo kusamala kuzachitidwa kuti zoipa sizizakhalepo. Ngati imwe kapena mwana wanu mwamva kuwawa koposa pathupi pomwe nsinganu inalowela, pitani pa cipatala ku kayanganiridwa.

Pojambula cithunzi cakubongo, thupi lanu limaunikidwa ndi kuwala kwa malaiti yamene ingathe kuvulaza dziwalo zina zamthupi kuwalaku kukacitidwa mobweredzabweredza.
Izi sizingacitike mukaunikidwa kwa nthawi imodzi cabe. *Ojambula cithunzi ca kubongo azaika singano ya pulastiki mu mitsempha yaku kwanja ndi kulasamo munkhwala wo onetsa bwino ngati muli ndi matenda mu bongo. Ngati muli ndi matenda okuza chitokhomiro kapena impso, dziwitsani ojambura kuti musalasidwe mankwala amenewa. Nthawi zocepekera zina mungathe kunsamva bwino pambuyo pokulasani munkwala uyu. Mwina mungave kuyabwa, kusapuma bwino komanso thupi kupya. Kumva thupi kupya kumacitika nthawi zonse koma sikumacita*

ngozi. Ngakale telo, dziwitsani ojambura mwamsanga, kuti apime ngati nkupitiriza ndi kujambula kapena ayi, kapena muzafunikira kusamaliridwa za kusamva bwino kwanu.
Ngati munalasidwapo munkwala uyu mukujambula koyamba nthawi ina, ndipo simunamve bwino pambuyo pake, dziwitsani ojambura popeza kuti, zingacitikensoZikatero, kujambula kungacitike kopanda singano ya munkhwala owonetsa bwino cithunzi

PHINDU KAPENA ZOPEZAMO
Inu kapena mwana wanu muzapezamo phindu uchokera mukufufuza uku chifukwa muzapimidwa ndikulandila mwaulele mankhwala yo chilitsa ku njoka zamumimba. Kuonjezerapo, kupyolera mwakujambura cithunzi ca ubongo wanu, muzadziwa ngati muli ndi tiana taminyoka mu ubongo wanu. *Kufufuza uku kuyembekedzedwa kubweletsa phinduso ku malo komwe imwe mukhala imwe chifukwa kuzadzetsa kuleta pansi matenda ku malo anu, ndiyeno kucepetsa m'pata wa anthu ena kutengako matenda awa. Imwe ndi ena otengako mbali mukufufuza uku simuzalandila malipilo ali onse komabe muzasamaliridwa mwa mayendedwe ndi mkhalidwe popita ku cipatala ku Chipata kapena ku Lusaka*

KUTETEZEDWA
Ngati mwapwetekedwa kapena muli ndi mafunso okhuza upwetekedwa kwa inu kapena kwa mwana wanu chifukwa chotengako mbali mu kufufuza uku, chonde onani a sing'anga pachipatala chakwanu uko omwe alikuthandizila kufufuza kwathu. Kutetedzewa kunakonzedwa ndi kuganidzidwapo kale ndipo muzasamalidwa mwaulele.

KUTETEZEDWA KWA ZACINSINSI ZA UMOYO WANU
Tizatha mwa mpavu zathu kusunga ci nsinsi pa umoyo wanu. Zonse zimene imwe kapena mwana wanu azapeleka mukufufuza uku zidzasungidwa mwachinsinsi ndi motetetdzewa mu malo obisika. Mapepala pomwe dzina lanu lalembedwa sadzaonewa ndi munthu ali onse komabe ofufuza, asin'ganga ndiponso anthu ena ocepekera omwe azafunikira kusunga cinsinsi monga oimilirako bungwe la Institute of Tropical Medicine. Ku saina kwanu kwapepala ili nkusimikiza kuti mukuvomera ndi makonzedwe awa osungilamo cinsinsi canu. Zothuluka mu kufufuza uku, zizafalitsidwa mu zolembedwa za kufufuza kwa umoyo ndipo ndiponso zizasungidwa mumalo osungiramo mbiri ya maiko onse.
Dzina lanu silizaonetsedwa paliponse muzolembedwa zothuluka mukufufuza uku.

BUNGWE LA ZAMACHITIDWE ABWINO OLOLEKA
Kufufuza uku kwavomeletsedwa ndi University of Zambia Biomedical Research Ethics Committee (Lusaka), Institutional Review Board of the Institute of Tropical Medicine, Antwerp (Belgium) ndi the Ethical Committee of the Antwerp University Hospital (Belgium). Awa mabungwe amayanganiranso ndi kuyesa kufufuza kumeneko macitidwe amapunziro ndikusimikiza kuti umoyo wa anthu otengamo mbali niwotetezeka kwa nthawi zonse.

KUTENGAKO MBALI KOZIPELEKA

Kutengako mbali kwainu kapena mwana wanu ndi kozipeleka kwathunthu. Ndi cosankha canu kutengamo mbali kapena ayi. Ndinu aufulu uchokamo panthawi iliyonse ya kufufuza uku, angakhale mutasaina kale pepala ya chivomezo chanu. Mungathe kuleka kutengamo mbali mukufufuza uku kopanda kupatsa cifukwa cake. Kuchita ichi sikuzakukhuzani inu kapena mwana wanu, anthu am'banja lanu kapenanso mudera mukhala inu munjira iliyonse.

Ofufuza nayenso angakuimikeni kukutengamo mbali mukufufuza uku nthawi iliyonse kopanda kukupempani atawoma kuti ndicho cingakukalireni bwino kapena kuti simusatira malangizo akufufuza uku ngakhale kuti munakumbutsidwa.

MUNTHU ODZIWITSA NGATI MULI NDI MAFUNSO

Ngati muli ndi mafunso okhuza utengako mbali kwanu inu mu kufufuza uku, kapena muganiza kuti mwapwetekwedwa chifukwa cah kufufuza uku, mukhoza kudwiwitsa pomwe pano, mukati kapena kumathero kwa kufufuza uku.

Dr. Gideon Zulu, Ministry of Health, Government of the Republic of Zambia. Cell: +260 976116556. E-mail: gideonzulu@yahoo.com

Kabemba Evans Mwape, Department of Clinical Studies, School of Veterinary Medicine, Box 32379, Lusaka. Tel: +260 211291515, Cell: +260 977819236. E-mail: kemwape@yahoo.com or evans.mwape@unza.zm

Research Ethics Committee, University of Zambia, Ridgeway Campus, P.O. Box 50110, Lusaka. Tel: 256067 Fax: 250753. E-mail: unzarec@zamtel.zm

7.3 Informed consent form in English

Federal Ministry of Education and Research

INSTITUTE OF TROPICAL MEDICINE ANTWERP

INFORMED CONSENT FORM

SOLID: Evaluation of a simple test for the diagnosis of pork tapeworm infection

Part which is destined only to the participant or his/her legal representative

I confirm that I have been informed about the study and a copy of the research participant information sheet and the Informed Consent Form was given to me. The study was explained to me in an understandable way and with sufficient information. There was enough time to consider the information and to ask questions, to which I have received satisfying answers.

I freely consent myself or consent for my child's participation and I will cooperate in the study examinations/activities, including donation of blood and stool samples, participating in clinical examinations, answering questionnaires and undergoing brain scanning. I am willing to give information concerning my medical history, use of medication and participation in other studies if any.

If I ever want to stop participation, even after signing the informed consent, I know I can do so.

I agree that my doctor and other healthcare professionals involved in my treatment are informed about my participation in this study.

☐ Yes / ☐ No: I agree that samples are stored for 10 years after completion of this study and may be used for future scientific research (in the field of pork tapeworm disease) (and I know that these samples will not be used for genetic testing).

To be completed by the participant (consent for adult participant and assent for participant less than 18 years old)

Date: ……………………………

Name of participant: ……………………………

Signature (or thumbprint) of participant: ……………………………

Note: the fingerprint replaces the signature, ONLY for illiterate participants (accompanied by a witness)

To be completed by the parent or legally authorized representative of the participant (in case the participant is less than 18 years old)

Date: ……………………………

Name of parent or legally authorized representative: ……………………………

In case there is no parent or legally authorized representative, consider asking to add the relationship of the guardian with the participant here as well.

Signature (or thumbprint) of parent or legally authorized representative: ……………………………

Note: the fingerprint replaces the signature, ONLY if the legal guardian is illiterate (accompanied by a witness)

E D C T P

Federal Ministry of Education and Research

INSTITUTE OF TROPICAL MEDICINE ANTWERP

To be completed by the witness (in case the participant/ parent or legally authorized representative is illiterate)

Date: ……………………………

Name of witness: ……………………………

Signature of witness: ……………………………

Note: If the participant (or parent or legally authorized representative) is unable to read and/or write, an impartial witness should be present during the informed consent discussion. After the written informed consent form is read and explained to the participant (or tutor) and after (s)he has orally consented to participation in the study, and has provided fingerprint, the witness should complete the name of the participant and add the date of fingerprint, and sign and personally date the consent form. By signing the consent form, the witness attests that the information in the consent form and any other written information was accurately explained to, and apparently understood by the participant (or parent or legally authorized representative), and that informed consent was freely given.

To be completed by the person obtaining the informed consent

I, undersigned, …………………………………………………………………………… confirm that I have informed the participant (and/or the parent or legally authorized representative) about all the relevant aspects of this study. I confirm that he/she has consented voluntarily to participate in the study.

Date: ……………………………

Signature: ……………………………

7.4 Informed consent form in Chewa

PEPALA YA CHIVOMEZO

SOLID: Kufufuza kwa kapimidwe kofewa mukuzindikira matenda ya minyoka zo chokera ku nkumba

Mbali yokhuza otengako mbali oka kapena owaimilila mwalamulo
Ndisimikiza kuti andifokotozela ndipo ndauzidwa zakufufuza uku ndiponso ndalandila pepala ya chivomezo changa. Ndapasiwanso mauthenga yokwanila ndithu kuzofunikila kudziwa, nthawi yofufuza ndipo pazosafunikila ndi zoipa zonse zokudza kufufuza. Kuonjezela, ndapatisdwa nthawi yokwanila ndithu kuganizilapo zimene ndaudzidwa ndiponso danga yapasidwa kutindifunseko mafunso, ngakhale mafunso yanga yonse yayankhidwa kukhutira kwanga.

Ndavomera mwaufulu kuti nditengeko mbali mukufufuza uku ndipo ndavomeranso ku machitidwe ndi zochitika zamene ndaphemphedwa, kuikilapo utengewako magazi ndi dothi (tuvi), mumafunso, ndi kujambulidwa cithunzi ca ubongo.

Ndine odzipereka ku lembetsa mbiri yanga ya umoyo, kugwiritsila ncito mankhwala ndikutengamo mbali mukufufuza kwinanso kukankalapo.
Nditafuna kuleka kutengamo mbali mukufufuza uku ngakhale kuti ndinasaina chivomerezo, ndikudziwa kuti ndingatero
Ndivomeretsa kuti asin'ganga ndi ena anchito akucipatala omwe amandiciritsa angauzidwe zakutengamo mbali kwanga mukufufuza uku.

☐ Inde / ☐ Ayi:
Ndivomera kuti magazi/dothi langa isungidwe kwa zaka kumi pakuti ikhatahndizile ufufuza mutsogolo (kufafana ndi kufufuza kwachitidwa mukufufuza kwa apa):

Chivomezo cha akulu otengako mbali (Kwa awo ali ndi dzaka zakubadwa zochuluka khumi ndi zisanu ndi zitathu):

Tsiku: ....

Dzina lawamkulu otengako mbali: ....

Kusaina kapena kusindikhiza kwa wamkulu wotengako mbali: ....

Note: the fingerprint replaces the signature, ONLY for illiterate participants (accompanied by a witness)

<u>Kuzulitsidwa ndi makolo kapena oyanganira mwana otengamo mbali</u> (Ngati mwana ali ndi zaka zobadwa zosakwanisa zaka kumi, zisanu ndi zitatu)

Tsiku: ....

Dzina la makolo kapena oimililako mwana: ....

In case there is no <u>parent or legally authorized representative</u>, consider asking to add the relationship of the guardian with the participant here as well.

Kusaina kwa makolo kapena oimililako mwana: ....

Note: the fingerprint replaces the signature, ONLY if the legal guardian is illiterate (accompanied by a witness)

Kuzulitsidwa ndi oimililako umboni (Ngati otengamo mbali kapena owaimililako sangathe kusaina)

Tsiku: ....

Dzina la oimililako umboni : ....

Kusaina kwa oimililako umboni : ....

Note: If the participant (or parent or legally authorized representative) is unable to read and/or write, an impartial witness should be present during the informed consent discussion. After the written informed consent form is read and explained to the participant (or tutor) and after (s)he has orally consented to participation in the study, and has provided fingerprint, the witness should complete the name of the participant and add the date of fingerprint, and sign and personally date the consent form. By signing the consent form, the witness attests that the information in the consent form and any other written information was accurately explained to, and apparently understood by the participant (or parent or legally authorized representative), and that informed consent was freely given.

<u>Kuzulitsidwa ndi otenga chivomerezo ici</u>

Ine, omwe ndasaina pansipa...ndisimikiza kuti ndinawafotokozera otengako mbali (makolo awo kapena owaimililako) zonse mbali zokhuza kufufuza uku.
Ndisimikiza kuti iwo avomera modzifunira kutengamo mbali mukufufuza uku

Tsiku: ....

Kusaina : ....

7.5 SOLID partners

Table 3: SOLID partners

Name	Affiliation	Field of expertise
Prof. S. Gabriël	Ghent University, Belgium	Parasitologist
Prof. P. Dorny	Institute of Tropical Medicine, Belgium	Parasitologist
Prof. M.V. Johansen	University of Copenhagen, Denmark	Parasitologist
Prof. I.K. Phiri	University of Zambia	Parasitologist
Prof. E. Bottieau	Institute of Tropical Medicine, Belgium	Medical doctor
Prof. Dr. Dr. A.S. Winkler	Technical University of Munich, Germany	Neurologist
Ms Veronika Schmidt	Technical University of Munich, Germany	Parasitologist
Ms K. Schou Møller	University of Copenhagen, Denmark	PhD student
Mr C. Mubanga	University of Zambia	PhD student
Dr P. Magnussen	University of Copenhagen, Denmark	Medical doctor
Dr O. Ciccone	University Teaching Hospital, Zambia	Neurologist
Dr K.E. Mwape	University of Zambia	Parasitologist
Dr I. Van Damme	Ghent University, Belgium	Statistician
Dr H. Ngowi	Sokoine University of Agriculture, Tanzania	Parasitologist
Dr G. Zulu	Ministry of Health, Zambia	Medical doctor
Dr E. Abatih	Ghent University, Belgium	Statistician
Dr C.R. Stensvold	Statens Serum Institut, Denmark	Parasitologist
Dr C. Trevisan	Institute of Tropical Medicine, Belgium	Parasitologist
Dr C. Chabala	University of Zambia	Neurologist
Dr B. Ngowi	National Institute for Medical Research, Muhimbili Medical Research Centre, Tanzania	Medical doctor
Dr B. Ndawi	Ministry of Health, Tanzania	Medical doctor

7.6 Detailed sample size calculations

Sample Size calculation for the SOLID project

Abatih Emmanuel

1st June 2017

Community-Level Study in Zambia

Background for Community-level Study

- We are interested in the performance of POC test for CC/T
- Reference test is best available method to establish presence of disease
- We will base our calculations on the expected sensitivity and specificity of the POC test for cysticercosis (CC) and Taeniasis, a fixed precision of the point estimate of d, and an estimated prevalance of a positive CC or Taeniasis in the study population of P%.

Requirements for Community Study

Some of the pieces of information we will need are:

- Se/Sp from previous publications in the zone or regions with similar characteristics
- Confidence Level
- Precision of estimations (d=+-10% of the truth)
- Anticipated apparent prevalence of CC and Taeniasis based on Antibody detection
- Obtain some estimates of sensitivity and specificity or state the minimal acceptable sensitivity or specificity
- Once these pieces of information are available, the minimum sample size (n_a) can be computed as:

$$n_a \geq \frac{Z^2_{(1-\alpha/2)} * S_N * (1 - S_N)}{d^2}$$

where S_N us the estimated sensitivty of POC test based on previous studies or expect knowlegde. From the example in Bonoo et al. 2010, to measure the Se of the POC test to within 10%, we require at least n_a samples that are positive by the Gold Standard test, so we will need to recruit $n_{ses} = \frac{n_a}{P}$ subjects from the communities to be able to have a total of n_a infected subjects with CC or T.

What we know

Based on unpublished data, the expected prevalence of Cysticercosis in the communities under consideration range between 33.5% and 38.5% with a modal value of 35%

For Cysticercosis, there are two main possibilities:

- For a single cyst: expected Se is 88% and expected Sp is 99% for the POC test
- For 2+ cysts: Expected Se is 93% and expected Sp is 99% for the POC test.

For Taeniasis (but this was the old one):

- Expected Se of POC test is 82% and expected Sp is 81%.

For both numbers around 95% are to be expected with the new strip, but this still has be tested (Schmidt, personal communication).

Sample sizes will be computed for several scenarios.

Adjustments

For this study, two kinds of adjustments are imminent: adjustments based on the sampling design scheme and those based on contigencies during sampling in the field.

Design effect

Based on the design of this study, households will be randomly sampled and all concenting individuals within the selected HH's will be included. Given that the number of individuals within households in these communities varies between 2 and up to 20 individuals (to be confirmed), a custering effect is expected. Adjustments for the clustering effect involve multiplying the estimated sample size by a value between 1.5 and 2 (reference).

Contigencies

It often happens that even after sending out notifications prior to visiting HHs, one or more members of a household are not present during sampling and even after repeated attempts, targeted subjects cannot be sampled. In order not to reduce the targeted sample size, amendements can be made to mitigate the effects of such unavoidable circumstances. This usually involves increasing the sample size by between 5% to 20% (reference).

Sample size calculation proper

Cysticerscosis

This function will calculate the sample size for a known value of Se and cysticercosis prevalence (prev_Cyst)

```
samplecommunbased<-function(se,prev_Cyst)
{
```

```
# calculate N

N=(1.96*1.96)*se*(1-se)/(0.05^2)
N

adjustedN=N/prev_Cyst
adjustedN


return(adjustedN)

}

#Compute optimal Sample size using the provided estimated values for POC and
CC prevalence: One cyst case
optimal_sample_size<-samplecommunbased(0.88,0.35)
print(optimal_sample_size)

## [1] 463.6262

designeffectN<-2*optimal_sample_size
Contigencyn<-0.15*optimal_sample_size

Total_N=designeffectN+Contigencyn
print(Total_N)

## [1] 996.7964

#Compute optimal Sample size using the provided estimated values for POC and
CC prevalence: 2+ cysts case

#Compute optimal Sample size using the provided estimated values for POC and
CC prevalence: One cyst case
##NOt sure if for two cysts, the prevalence is the same
optimal_sample_size2<-samplecommunbased(0.93,0.35)
print(optimal_sample_size2)

## [1] 285.815

designeffectN2<-2*optimal_sample_size2
Contigencyn2<-0.15*optimal_sample_size2

Total_N2=designeffectN2+Contigencyn2
print(Total_N2)

## [1] 614.5023
```

The optimal sample size based on a single cyst is 997 and based on 2+ cysts the optimal sample size is 615 subjects.

Simulations

Here we present results of a simulation study wherein the Sample sizes were computed for varying values of the Se and the expected prevalence of CC. The min and max of all the values of the sample size computed will be used as a range of plausible values of the sample size.

```
set.seed(201705)
sim_runs=10000
est_sample_cyst<-list()
est_sample_cyst2<-list()
sample_cyst<-rep(NA, sim_runs)
sample_cyst2<-rep(NA, sim_runs)
##Single cyst: sens 88% spec 99%
#we sample values of the se ranging from 0.80 to 0.96 from a uniform distribu
tion
senspoc_cyst<-runif(sim_runs, 0.80,0.96)

##2+ cysts: sens 93% spec 99%
#we sample values of the se ranging from 0.89 to 0.98 from a uniform distribu
tion
senspoc_cyst2<-runif(sim_runs, 0.89,0.97)

#we sample values of the expected prevalence of CC from 0.335 to 0.385 from a
uniform distribution
ant_prev_cyst<-runif(sim_runs, 0.335,0.385)


for(i in 1:sim_runs)
{sample_cyst[i]<-2*samplecommunbased(senspoc_cyst[i],ant_prev_cyst[i]) + 0.15
*samplecommunbased(senspoc_cyst[i],ant_prev_cyst[i])
est_sample_cyst[[i]]=c(round(i,0),senspoc_cyst[i],ant_prev_cyst[i], sample_cy
st[i])
}
results<-matrix(unlist(est_sample_cyst),,4, byrow=TRUE)
resultsintable<-as.data.frame(results)
names(resultsintable)<-c("Sim_ID","Se_POC", "Prev_CC", "Adjusted_N")
summary(resultsintable$Adjusted_N)

##    Min. 1st Qu.  Median    Mean 3rd Qu.    Max.
##   331.4   680.2   976.0   955.5  1239.0  1574.0

head(resultsintable[order(resultsintable$Se_POC),])

##       Sim_ID    Se_POC   Prev_CC Adjusted_N
## 1973    1973 0.8000229 0.3446870   1533.445
## 872      872 0.8000233 0.3412680   1548.806
## 9814    9814 0.8000289 0.3415430   1547.527
```

```
## 3668   3668 0.8000794 0.3673176   1438.664
## 4362   4362 0.8000838 0.3623794   1458.245
## 9986   9986 0.8001044 0.3710166   1424.187
```

```
for(i in 1:sim_runs)
{sample_cyst[i]<-2*samplecommunbased(senspoc_cyst2[i],ant_prev_cyst[i]) + 0.1
5*samplecommunbased(senspoc_cyst2[i],ant_prev_cyst[i])
est_sample_cyst2[[i]]=c(round(i,0),senspoc_cyst2[i],ant_prev_cyst[i], sample_
cyst[i])
}
results2<-matrix(unlist(est_sample_cyst2),,4, byrow=TRUE)
resultsintable2<-as.data.frame(results2)
names(resultsintable2)<-c("Sim_ID","Se_POC", "Prev_CC", "Adjusted_N")
summary(resultsintable2$Adjusted_N)
```

```
##    Min. 1st Qu.  Median    Mean 3rd Qu.    Max.
##   251.3   435.5   595.8   592.7   749.9   961.4
```

```
head(resultsintable2[order(resultsintable2$Se_POC),])
```

```
##      Sim_ID    Se_POC   Prev_CC Adjusted_N
## 8729   8729 0.8900133 0.3536989   914.3523
## 860     860 0.8900151 0.3501541   923.5953
## 2497   2497 0.8900428 0.3618341   893.5842
## 7614   7614 0.8900614 0.3362521   961.4256
## 4834   4834 0.8900630 0.3492101   925.7390
## 8679   8679 0.8900684 0.3814130   847.5418
```

The results from this simulation study (10k runs) indicated that, sample sizes ranging from 332 to 1574 subjects are valid for this study. Thus a minimum of 332 and a maximum of 1574 individuals will have to be sampled from HH's within the communities under investigation to be able to precisely assess the performance of the POC test for CC (one cyst case) after making provisions for design effects and other contigencies. The optimal sample size for this study is 997 individuals. For the case with 2+ cysts, the range of plausible values was found to be : (252 to 962) with an opitimal value of 615 subjects. So it can be concluded that with at least one cyst, the minimum required sample size is 332 and the maximum is 1574 for the case of CC.

For Taeniasis

For Taeniasis, information is available on test performance but no prevalence data available based on antibody detection. An expected prevalence of 5.9% was therefore assumed.

Compute optimal Sample size using the provided estimated values for POC and Taeniasis assumed prevalence

```
optimal_sample_size_T<-samplecommunbased(0.82,0.059)
print(optimal_sample_size_T)
```

```
## [1] 3844.204

designeffectNT<-2*optimal_sample_size_T
ContigencynT<-0.15*optimal_sample_size_T

Total_NT=designeffectNT+ContigencynT
print(Total_NT)

## [1] 8265.04
```

Simulation study: varying the prevalence and the POC test se

```
set.seed(120175)
sim_runs=10000
est_sample_T<-list()

sample_T<-rep(NA, sim_runs)

##Single cyst: sens 88% spec 99%
#we sample values of the se ranging from  from a uniform distribution
senspoc_T<-runif(sim_runs, 0.78,0.98)

#we sample values of the expected prevalence of CC from  from a uniform distr
ibution
ant_prev_T<-runif(sim_runs, 0.04,0.10)


for(i in 1:sim_runs)
{sample_T[i]<-2*samplecommunbased(senspoc_T[i],ant_prev_T[i]) + 0.15*sampleco
mmunbased(senspoc_T[i],ant_prev_T[i])
est_sample_T[[i]]=c(round(i,0),senspoc_T[i],ant_prev_T[i], sample_T[i])
}
results_T<-matrix(unlist(est_sample_T),,4, byrow=TRUE)
resultsintable_T<-as.data.frame(results_T)
names(resultsintable_T)<-c("Sim_ID","Se_POC", "Prev_Taeniasis", "Adjusted_N")
summary(resultsintable_T$Adjusted_N)

##    Min. 1st Qu.  Median    Mean 3rd Qu.    Max.
##   671.8  3011.0  4877.0  5108.0  6723.0 14050.0

head(resultsintable_T[order(resultsintable_T$Se_POC),])

##      Sim_ID     Se_POC Prev_Taeniasis Adjusted_N
## 8377   8377 0.7800196     0.08497556   6671.233
## 1989   1989 0.7800220     0.09948410   5698.271
## 6501   6501 0.7800513     0.09838438   5761.413
## 6508   6508 0.7800535     0.08164563   6942.551
## 2368   2368 0.7800886     0.07553757   7503.074
## 9399   9399 0.7800971     0.04054130  13979.530
```

The results from this simulation study (10000 runs) for the case of Taeniasis indicated that, sample sizes ranging from 672 to 14050 subjects are valid for this study. Thus a minimum of 672 and a maximum of 14050 individuals will have to be sampled from HH's within the communities under investigation to be able to precisely assess the performance of the POC test for Taeniasis after making provisions for design effects and other contigencies.

An intermittent or mid-term analysis will be done after 700 samples have been collected in in order to re-estimate the same size and make adjustments where necessary.

References

- Banoo S, Bell D, Bossuyt P, Herring A, Mabey D, Poole F, Smith PG, Sriram N, Wongsrichanalai C, Linke R, O'Brien R, Perkins M, Cunningham J, Matsoso P, Nathanson CM, Olliaro P, Peeling RW, Ramsay A, TDR Diagnostics Evaluation Expert Panel. Evaluation of diagnostic tests for infectious diseases: general principles. Nat. Rev. Microbiol. 2010: 8(12 Suppl): S16-28. doi:10.1038/nrmicro1523
- Chota A., Sikasunge C.S., Makungu C., Mwape K.E., Simuunza M., Chembensofu M., Phiri I.K. (2014). Prevalence of Taenia Solium Infections In Humans And Pigs In Rural Communities Of Monze District Of Southern Zambia. University of Zambia Journal of Science and Technology. 18(182), 9-18.
- KP Suresh and S Chandrashekara: Sample size estimation and power analysis for clinical research studies. J Hum Reprod Sci. 2012 Jan-Apr; 5(1): 7-13.
- Mwape KE, Phiri IK, Praet N, Dorny P, Muma JB, et al. (2014). Study and ranking of determinants of Taenia solium infections by classification tree models. Am J Trop Med Hyg. 92(1):56-63. doi: 10.4269/ajtmh.13-0593
