# Supplementary material for: Challenges Encountered When Evaluating an Antibody-Detecting Point-of-Care Test for Taeniosis in an Endemic Community in Zambia: A Prospective Diagnostic Accuracy Study
Source: Diagnostics (Basel). 2021 Nov 4;11(11):2039. doi: 10.3390/diagnostics11112039 (PMC8625551; doi:10.3390/diagnostics11112039)
Supplement: Supplementary file 1 [file diagnostics-11-02039-s001.zip › diagnostics-1431851-supplementary/TS POC T supplementaryfiles/File S3_STARD Checklist_26092021.pdf]

File S3. STARD checklist

Section & Topic	No	Item	Section (page)	Explanation
TITLE OR ABSTRACT				
	1	Identification as a study of diagnostic accuracy using at least one measure of accuracy<br>(such as sensitivity, specificity, predictive values, or AUC)	Title (p**Error! Bookmark not defined.**)<br>Abstract (p**Error! Bookmark not defined.**)<br>Keywords (p**Error! Bookmark not defined.**)	"**Error! Reference source not found.**"<br>Keywords: "sensitivity", "specificity"
ABSTRACT				
	2	Structured summary of study design, methods, results, and conclusions<br>(for specific guidance, see STARD for Abstracts)	Abstract (p**Error! Bookmark not defined.**)	The abstract was written according to the STARD guidelines for abstracts
INTRODUCTION				
	3	Scientific and clinical background, including the intended use and clinical role of the index test	Introduction (p8)	Intended use: Epidemiologically, the purpose of the TS POC T is to identify spots of active human transmission while clinically is to identify infected individuals who need to be treated.<br>Clinical role: new test: there is currently a lack of well-performing, field-deployable, easy-to-use diagnostic tests to either identify infected people
	4	Study objectives and hypotheses	**Error! Reference source not found.** (p**Error! Bookmark not defined.**)	The TS POC performance was evaluated …………diagnostic accuracy outcomes, sensitivity, specificity and predictive values of the community-based TS POC T evaluation and challenges encountered
METHODS				
Study design	5	Whether data collection was planned before the index test and reference standard were performed (prospective study) or after (retrospective study)	**Error! Reference source not found.** (p8)	Prospective study using a two-stage design: after recruitment, all participants were tested using the index test, after which a subset was selected for reference testing
Participants	6	Eligibility criteria	**Error! Reference source not found.** (p**Error! Bookmark not defined.**)	To participate in the study, participants had to live in the study area, be 10 years of age or older, give written informed consent and express willingness to participate in all study aspects such as, getting tested, provision of blood and stool samples, as well as going for CT scan if and when it was required. Those who were reported severely ill, pregnant or visiting the study area were not recruited for the study.
	7	On what basis potentially eligible participants were identified<br>(such as symptoms, results from previous tests, inclusion in registry)	**Error! Reference source not found.** (p**Error! Bookmark not defined.**)	Study participants were recruited from randomly selected households. The eligibility criteria were not strict, in order to obtain a representative sample from the population.

	8	Where and when potentially eligible participants were identified (setting, location and dates)	**Error! Reference source not found.** (p**Error! Bookmark not defined.**)	Recruitment was done in four communities in Sinda district, Eastern Province of Zambia. The recruitment took place from December 2017 to June 2019.
	9	Whether participants formed a consecutive, random or convenience series	**Error! Reference source not found.** (p**Error! Bookmark not defined.**)	Households were randomly selected and all consenting eligible individuals in these households were recruited.
Test methods	10a	Index test, in sufficient detail to allow replication	**Error! Reference source not found.** (p**Error! Bookmark not defined.**)	TS POC T test strip: a lateral flow assay using the recombinant protein rES33 to detect antibodies against the tapeworm
	10b	Reference standard, in sufficient detail to allow replication	**Error! Reference source not found.** (p**Error! Bookmark not defined.**)	Three reference tests were used: rES33 EITB, copro multiplex PCR and copro Ag ELISA: (1) rES33 EITB with a few modifications, the conjugate was used at a dilution of 1:1000 while the 3, 3'-Diaminobenzidine (DAB, 10mg Sigma Aldrich) and the hydrogen peroxide was used according to manufacturer's instructions [1,2]; (2) copro multiplex PCR for *T. solium* (copro PCR) [3] and (3) copro Ag ELISA [4], as modified [5].
	11	Rationale for choosing the reference standard (if alternatives exist)	Introduction (p**Error! Bookmark not defined.**)	Different tests, including the ones that were used in this study, are described in detail in the introduction
	12a	Definition of and rationale for test positivity cut-offs or result categories of the index test, distinguishing pre-specified from exploratory	**Error! Reference source not found.** (p**Error! Bookmark not defined.**)	A pink line on the test line level was positive; the absence of the line was negative
	12b	Definition of and rationale for test positivity cut-offs or result categories of the reference standard, distinguishing pre-specified from exploratory	**Error! Reference source not found.** (p**Error! Bookmark not defined.**1)	Only pre-specified cut-off values were used. References to the papers are included in the text and more details can also be found in [6].
	13a	Whether clinical information and reference standard results were available to the performers/readers of the index test	**Error! Reference source not found.** (p**Error! Bookmark not defined.**)	Reference test results were not available when the index test was performed (index test was performed before the reference tests)
	13b	Whether clinical information and index test results were available to the assessors of the reference standard	**Error! Reference source not found.** (p**Error! Bookmark not defined.**)	Laboratory personnel was blinded to the TS POC T result. One researcher (CM), who was part of the field recruitment also participated in analysis with the copro Ag ELISA (partially blinded)
Analysis	14	Methods for estimating or comparing measures of diagnostic accuracy	**Error! Reference source not found.** (p**Error! Bookmark not defined.**)	A Bayesian analysis with probabilistic constrains was used
	15	How indeterminate index test or reference standard results were handled	**Error! Reference source not found.** (p**Error!**	Indeterminate index test results were excluded (see also Fig 1)

			**Bookmark not defined.**3)	
	16	How missing data on the index test and reference standard were handled	**Error! Reference source not found.** (p**Error! Bookmark not defined.**3)	Only complete cases were used in the analysis (see also Fig 1)
	17	Any analyses of variability in diagnostic accuracy, distinguishing pre-specified from exploratory	**Error! Reference source not found.** (p**Error! Bookmark not defined.**3)	The agreement between different test is an exploratory outcome of the study
	18	Intended sample size and how it was determined	**Error! Reference source not found.** (p**Error! Bookmark not defined.**)	The calculated target sample size of 1200 participants (to obtain a desired precision of 10% [6]
RESULTS				
Participants	19	Flow of participants, using a diagram	Fig 1 p15	
	20	Baseline demographic and clinical characteristics of participants	**Error! Reference source not found. Error! Reference source not found.** (p14-**Error! Bookmark not defined.**)	
	21a	Distribution of severity of disease in those with the target condition	NA	People were recruited from the community. Taeniosis is usually asymptomatic (see introduction). Disease severity was not recorded.
	21b	Distribution of alternative diagnoses in those without the target condition	NA	People were recruited from the community. Alternative diagnoses were not recorded.
	22	Time interval and any clinical interventions between index test and reference standard	**Error! Reference source not found.** (p9)	There was no time interval between TS POC T testing and sample collection nor any clinical intervention
Test results	23	Cross tabulation of the index test results (or their distribution) by the results of the reference standard	Table 1 p16 Fig 1 (flow) p15	Result combination between TS POC T and reference tests
	24	Estimates of diagnostic accuracy and their precision (such as 95% confidence intervals)	Table 2 **Error! Reference source not found.** (p**Error! Bookmark not defined.**7)	The estimated sensitivity and specificity for the TS POC T test were 38% (95% CI: 5-93%) and 99% (95% CI: 98-100%), respectively.
	25	Any adverse events from performing the index test or the reference standard	**Error! Reference source not found.** (p**Error! Bookmark not defined.**)	No adverse events were recorded.
DISCUSSION				
	26	Study limitations, including sources of potential bias, statistical uncertainty, and generalisability	**Error! Reference source not found.** (**Error! Bookmark not defined.**)	Sources of potential bias: The analysis failed to account for the impact of the TS POC CC result on the sampling (P20, line 399) and the refusing population (in case they would have a different disease spectrum (P23, line 470)) Statistical uncertainty: explanations for wide credibility intervals for sensitivity are given

				in the second paragraph of the discussion P19, 21
	27	Implications for practice, including the intended use and clinical role of the index test	**Error! Reference source not found.** (**Error! Bookmark not defined.**)	The low sensitivity makes the test in its current form unfit for purpose (P20, line 385)
OTHER INFORMATION				
	28	Registration number and name of registry	**Error! Reference source not found.** (p**Error! Bookmark not defined.**)	The SOLID project was registered under the Pan African Clinical Trial Registry PACTR20171200278889
	29	Where the full study protocol can be accessed	S2 file	The protocol is included as supporting information of this manuscript
	30	Sources of funding and other support; role of funders	Financial disclosure section	This work was funded by the European & Developing Countries Clinical Trials Partnership (EDCTP; grant number DRIA2014-308 SOLID) and the German Federal Ministry of Education and Research (BMBF; grant number: 01KA1617) within the research grant "Evaluation of an antibody detecting point-of-care test for the diagnosis of *Taenia solium* taeniosis and (neuro)cysticercosis in communities and primary care settings of highly endemic, resource-poor areas in Tanzania and Zambia, including training of – and technology transfer to the Regional Reference Laboratory and health centers (SOLID)". The funders had no role in this study.
